# Landscape connectivity among remnant populations of guanaco (*Lama guanicoe* Müller, 1776) in an arid region of Chile impacted by global change

**DOI:** 10.7717/peerj.4429

**Published:** 2018-03-02

**Authors:** Mara I. Espinosa, Nicolas Gouin, Francisco A. Squeo, David López, Angéline Bertin

**Affiliations:** 1 Departamento de Biología, Facultad de Ciencias, Universidad de La Serena, La Serena, Chile; 2 Centro de Estudios Avanzados en Zonas Áridas, La Serena, Chile; 3 Instituto de Investigación Multidisciplinar en Ciencia y Tecnología, Universidad de La Serena, La Serena, Chile; 4 Instituto de Ecología y Biodiversidad, Santiago, Chile

**Keywords:** Animal movement, Circuitscape, Guanaco, Habitat modelling, *Lama guanicoe*, Functional connectivity

## Abstract

Connectivity between populations plays a key role in the long-term persistence of species in fragmented habitats. This is of particular concern for biodiversity preservation in drylands, since water limited landscapes are typically characterized by little suitable habitat cover, high habitat fragmentation, harsh matrices, and are being rapidly degraded at a global scale. In this study, we modelled landscape connectivity between 11 guanaco *Lama guanicoe* populations in Chile’s arid Norte Chico, a region that supports the last remnant coastal populations of this emblematic herbivore indigenous to South America. We produced a habitat suitability model to derive a regional surface resistance map, and used circuit theory to map functional connectivity, investigate the relative isolation between populations, and identify those that contribute most to the patch connectivity network. Predicted suitable habitat for *L. guanicoe* represented about 25% of the study region (i.e., 29,173 km^2^) and was heterogeneously distributed along a continuous stretch along the Andes, and discontinuous patches along the coast. As a result, we found that high connectivity current flows in the mid and high Andes formed a wide, continuous connectivity corridor, enabling connectivity between all high Andean populations. Coastal populations, in contrast, were more isolated. These groups demonstrate no inter-population connectivity between themselves, only with higher altitude populations, and for two of them, animal movement was linked to the effectiveness of wildlife crossings along the Pan-American highway. Our results indicate that functional connectivity is an issue of concern for *L. guanicoe* in Chile’s Norte Chico, implying that future conservation and management plans should emphasize strategies aimed at conserving functional connectivity between coastal and Andean populations, as well as the protection of habitat patches likely to act as stepping stones within the connectivity network.

## Introduction

Understanding and managing connectivity has become a key concern for the conservation of biological populations and communities in the face of rapid habitat loss and fragmentation driven by anthropogenic and climate change effects (Mitchell, Bennett & Gonzalez, 2013; Correa Ayram et al., 2014; Riordan et al., 2015; Dilts et al., 2016). By facilitating genetic exchange between habitat patches, connectivity plays a fundamental role in the long-term persistence of species in fragmented habitats (Fahrig & Merriam, 1994; Coughenour, 2008; Kindlmann & Burel, 2008). This is of particular concern in highly fragmented landscapes, where habitat loss results in exponential increases in patch distances, and thus dramatically intensifies habitat isolation (Andrén, 1994). Connectivity depends on both the proportion of suitable habitat across the landscape as well as the permeability of the surrounding matrix. According to empirical and theoretical evidence, patch isolation negatively impacts population size and species richness in birds and mammals when suitable habitat cover is low (Andrén, 1994; Radford, Bennett & Cheers, 2005). It is believed to become a significant factor when the amount of suitable habitat in the landscape falls below 10–30% (Andrén, 1994; Betts et al., 2006), although this threshold may be greatly underestimated for many species (Mönkkönen & Reunanen, 1999). The surrounding matrix, on the other hand, may either facilitate or hinder patch connectivity by determining the permeability of the landscape to species movement.

**Figure 1:**
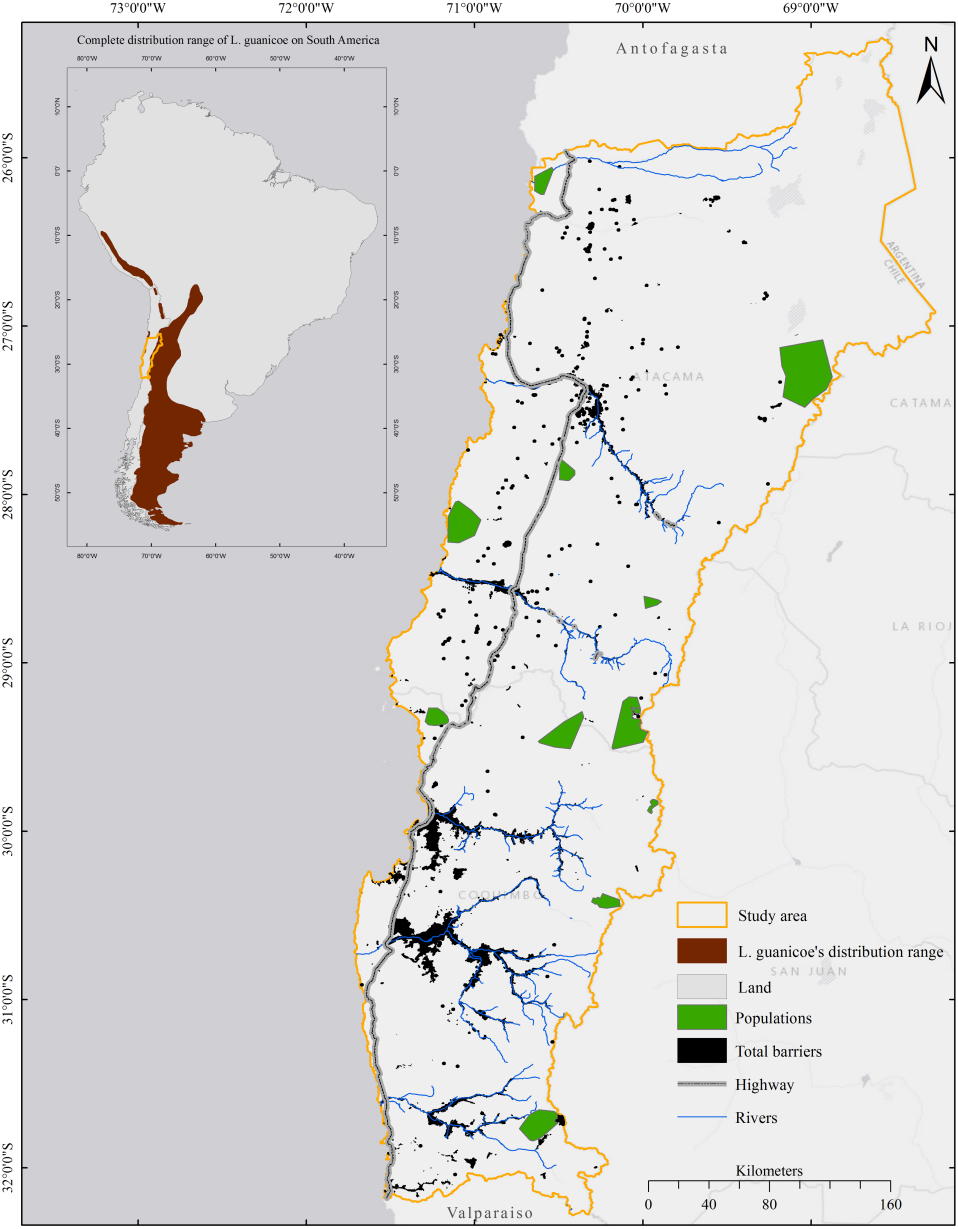
Distribution range of *Lama guanicoe* according to Baldi et al. (2016) and location of the study area.

The guanaco, *Lama guanicoe* (Artiodactyla, Camelidae), is an emblematic herbivore indigenous to South America, occurring in Peru, Bolivia, Chile, Paraguay and Argentina (Fig. 1). *L. guanicoe* has disappeared from 75% of its original range during the last century due to anthropogenic habitat disturbance and overhunting (Cunazza, Puig & Villalba, 1995; Ceballos & Ehrlich, 2002; Baldi et al., 2016), and its distribution appears discontinuous along its northern range (see map in Marín et al., 2013). While still classified as a species of least concern at the continental scale (Baldi et al., 2016), its conservation status varies across its distribution range. In Chile, *L. guanicoe* is considered vulnerable, particularly in the north of its distribution, where it occurs in small and isolated populations (Marín et al., 2013; González & Acebes, 2016). Because guanacos need expansive areas (Baldi et al., 2016), movements between habitat patches may not only be necessary to maintain effective population sizes, and thereby the evolutionary potential and long-term survival of the species, but also for individuals to meet their essential needs. *L. guanicoe* is able to travel long distances, which is demonstrated by large home-ranges (up to 600 km^2^ in the Payunia reserve, western Argentina) and the extensive migratory movements that have been recorded (i.e., up to 160 km) (Novaro, 2010). Human development and pressures may act to limit movement in this species, however, threatening populations in areas where resources are heterogeneously distributed, scarce, or transitory (Hobbs et al., 2008).

In this study, we modelled functional connectivity for *L. guanicoe* over the semi-arid region of Chile’s Norte Chico (between 26°S and 32°S latitude), home to the last remnant populations on the Pacific coast, comprising both cordilleran and pre-cordilleran groups. Chile’s Norte Chico is one of the most environmentally fragile areas in South America (Downing, 1994), and has experienced accelerated rates of desertification exacerbated by human activities (i.e., mining, agriculture, livestock production and tourism) and the overexploitation of scarce natural resources such as scrublands and seasonal grasslands for firewood collection and livestock grazing (Campos-Ortega & Jorquera-Jaramillo, 2008; Estevez et al., 2010). No information regarding movement and dispersal by individuals between populations in this region is available. Although distances between coastal and cordilleran populations (<200 km) are within the movement range of this species, various landscape features potentially obstruct displacement across the landscape, and in particular may result in isolation of coastal populations. These include several human settlements, which are interspersed among the coastal populations, and a fenced four-lane highway that transects the study zone from north to south, effectively segregating inland and coastal populations. This connectivity issue has been identified in a recent regional scale habitat modelling study (González et al., 2013), which predicted large areas of unsuitable habitat between coastal and inland populations that potentially act as a biogeographical barrier. Given that the predictive power of large scale models is often impaired due to local niche variation (Osborne & Suárez-Seoane, 2002; Murphy & Lovett-Doust, 2007), regional-scale studies are required to evaluate possible effects of local landscape characteristics on animal movement. The goals of our study were thus to identify potential inter-population migration routes between *L. guanicoe* populations in the region, assess the degree of connectivity of coastal populations in particular, and to identify habitat patches that most contribute to the network connectivity of the study area. To accomplish these objectives, we applied a resistance-surface-based connectivity modelling approach, first generating a surface resistance layer using a regional-scale habitat surface model, and finally mapping functional connectivity based on circuit-theoretic connectivity models.

## Materials and Methods

### Study area

**Figure 2:**
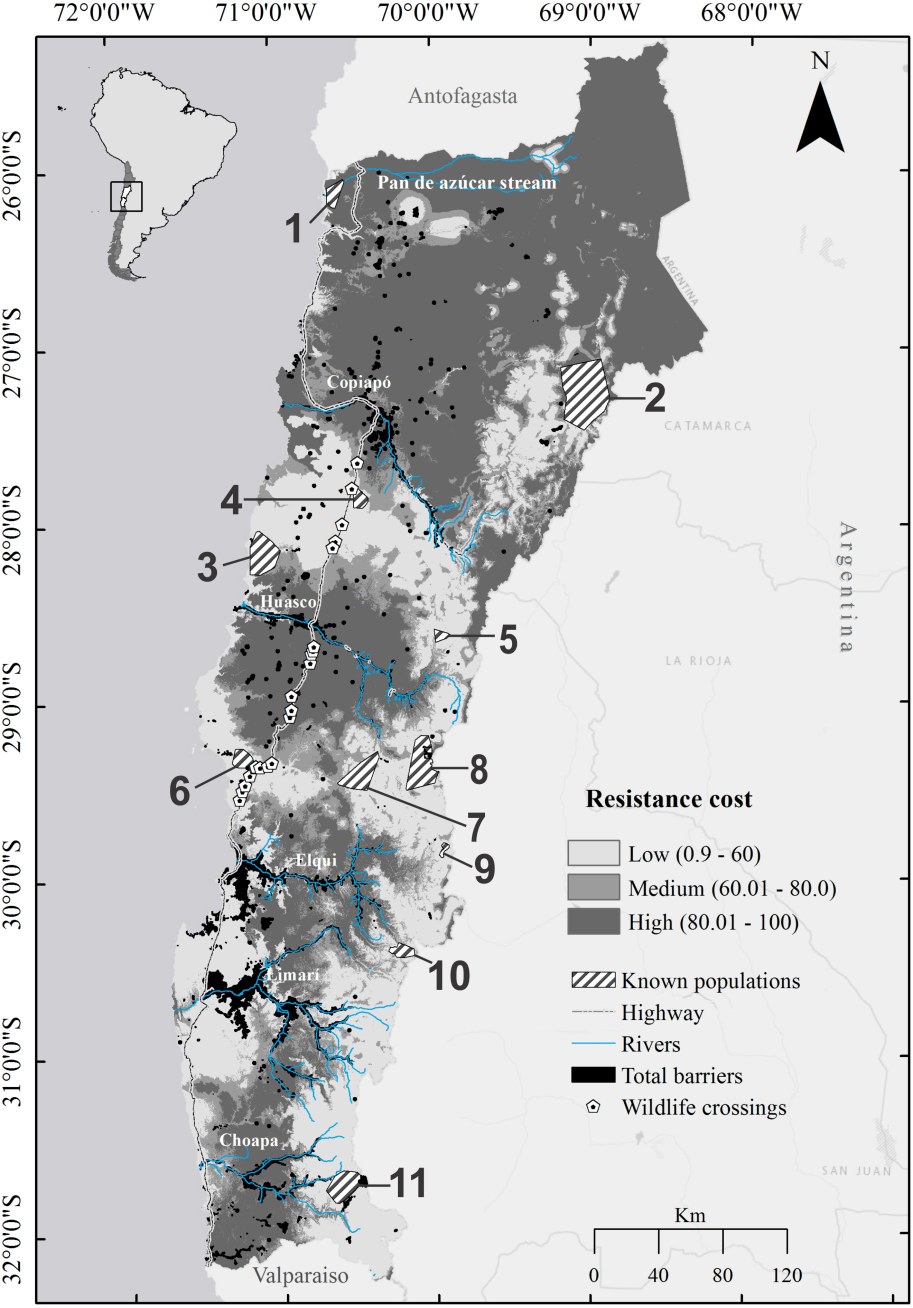
Map of landscape resistance for the guanaco *Lama guanicoe* in Chile’s Norte Chico. Resistance values were estimated by inverting and rescaling the habitat suitability values, generated with MaxEnt, to a continuous scale from 1 (low resistance/high suitability for dispersal) to 100 (high resistance/low suitability for dispersal). Numbers represent patches of habitat corresponding to the guanaco populations (**1**, Pan de Azúcar National Park; **2**, Nevado Tres Cruces National Park; **3**, Llanos de Challe National Park; **4**, Oso Negro sector; **5**, El Morro; **6**, Los Choros; **7**, Calvario stream; **8**, Tres Quebradas river; **9**, El Tambo stream; **10**, Estero Derecho nature sanctuary; **11**, Pelambres area).

The Norte Chico region in Chile is located between 26°S and 32°S (Fig. 1). It spans about 115,756 km^2^ and encompasses five hydrologic basins (Fig. 2). It is characterized by steep topography, with altitude increasing from zero to ∼5,000 masl within a distance of only 200 km inland from the coast (Squeo, Arancio & Gutiérrez, 2008; Zabala & Trigos, 2009). The climate is predominantly arid, although average temperature, precipitation, and relative humidity vary strongly according to both altitude and latitude (Juliá, Montecinos & Maldonado, 2008). The vegetation is composed of xeric shrublands, woody-stemmed shrubs, spiny scrubs and columnar and spherical cacti patchily distributed within an arid matrix (Novoa, Tracol & López, 2008). Evergreen trees and shrublands dominate slopes, while elevations >2,800 masl are dominated by cushion-forming plants, xeric herbs adapted to low temperatures, and high Andean wetland plant species (Squeo et al., 2006; Arancio & Marticorena, 2008).

Chile’s Norte Chico region is one of the most environmentally fragile areas in South America (Downing, 1994); important climatic changes over the last century (Fiebig-Wittmaack et al., 2012) have resulted in accelerated rates of desertification in the region, exacerbated by human activities (i.e., mining, agriculture, livestock production and tourism) and the overexploitation of scarce natural resources such as scrublands and seasonal grasslands for firewood collection and livestock grazing (Campos-Ortega & Jorquera-Jaramillo, 2008; Estevez et al., 2010). The road network includes a coastal highway with four-lanes that crosses the region from north to south and various secondary roads.

### *Lama guanicoe* ocurrence data

We collated presence data throughout the study zone, which includes eleven recorded *L. guanicoe* populations, comprising three coastal (Pan de Azúcar National Park (1), Llanos de Challe National Park (3) and Los Choros (6)), two mid-slope (El Calvario stream (7) and Oso Negro sector (4)) and six high altitude populations (Nevado Tres Cruces National Park (2), El Morro Private Protection Area (5), Tres Quebradas River Area of high conservation value (8), El Tambo stream (9), Estero Derecho Private Protection Area and Nature Sanctuary (10), and Pelambres Private Area (11)) (Fig. 2). While there have been no reports of any other coastal or mid elevation populations in the northern or southern extents of the study area, eastern Andean populations do exist on the Argentinean side. Both genetic (Marín et al., 2013) and telemetric (ULS, 2012-2015, unpublished data) data suggests that dispersal among the Chilean and Argentinean populations can occur.

For populations 3, 6, 7, and 8, we took GPS coordinates of fresh fecal deposits in each season from 2012 to 2014. Additionally, we considered geolocation data registered at ten day intervals between December 2012 and December 2015 for three collared individuals, one in Los Choros (6) (GPS-GSM Ecotone collars), and two in Tres Quebradas River Area (8) (Argos satellite telemetry). Finally, we completed our occurrence database by incorporating information from published sources (González et al., 2013; Bonacic et al., 2014) and observations recorded by researchers of the Department of Biology at the University of La Serena. The occurrence data were essentially recorded between 2002 and 2015 (Table S1), except for the El Tambo stream sector which were observed between 1994 and 2008 (Table S1). To minimize spatial autocorrelation issues, we filtered the records using ENMtools (Warren, Glor & Turelli, 2008; Warren, Glor & Turelli, 2010), so that the distance between any two presence data was at least 500 m. The final occurrence dataset used in suitability modeling included 937 spatially unique records, of which 12.8% were obtained from collared individuals (Table S1).

### Environmental variables

Eight environmental variables were considered for the ecological niche models (Table 1), based on *a priori* expectations of their influence on guanaco populations. All raster maps were prepared and analyzed with a ∼90 m spatial resolution. The landscape variables included characteristics of topography and vegetation cover. Topographic factors were considered because available evidence indicates that guanacos tend to prefer mountainous areas with high and medium slopes (Travaini et al., 2007; Acebes et al., 2010; Pedrana et al., 2010). We derived elevation and slope layers from Shuttle Radar Topography Mission digital elevation data (SRTM, Farr et al., 2007), with a spatial resolution of 3 arc-seconds (http://srtm.csi.cgiar.org), using Spatial Analyst in ArcGIS 10.2.1 (ESRI, 2014). In addition, we calculated the surface roughness as recommended by Riley (1999) using the Geomorphometry and gradient metrics toolbox version 2.0 (Evans et al., 2014) for ArcGIS. Since local vegetation plays a determinant role in habitat selection by animals at fine spatial scales (Kotliar & Wiens, 1990; Chetkiewicz, Clair & Boyce, 2006), including guanacos (Puig et al., 2008), we derived vegetation cover types from the classification of Chilean vegetation communities (Luebert & Pliscoff, 2006). This information is summarized in Table S2. Access to water and statutory protected status are both considered to positively influence *L. guanicoe’s* survival, and layers representing distance to both water sources and protected areas were accordingly generated for use in habitat suitability modelling. Water sources and protected area boundaries were identified based on the National Wetlands Inventory (MMA, 2011), and Coquimbo and Atacama red books (Squeo, Arancio & Gutiérrez, 2001; Squeo, Arancio & Gutiérrez, 2008), respectively. We used distance to both human settlements and roads as proxies for human disturbances. The roads layer was generated based on data from the Ministerio de Obras Públicas de Chile (MOP, 2013). Only paved roads were included. Finally, the distance to human settlements raster was produced based on the Open Street Map database (https://www.openstreetmap.org).

**Table 1:** Environmental variables used for habitat suitability modeling of *Lama guanicoe* in Chile’s Norte Chico.

GIS data layer	Description	Potential relevance for guanacos	Reference
Elevation	Altitude above sea level	Preference for mountainous areas	Travaini et al. (2007) and Acebes et al. (2010)
Roughness	Surface roughness	Surface roughness influences terrestrial animal movement	Dickson, Jenness & Beier (2005)
Slope	Rate of maximum change in *z*-values	Preference for high and medium slopes	Travaini et al. (2007) and Acebes et al. (2010)
Distance to urban areas	Euclidean distance to nearest urban area	Negative impact of dog attacks, poaching, competition with livestock and human activities in general	Vargas, Bonacic & Moraga (2016)
Distance to water bodies	Euclidean distance to nearest water bodies	Water is necessary for survival and physiological functions	Lautier, Dailey & Brown (1988), Packard (1991) and Prenda, López-Nieves & Bravo (2001)
Distance to roads	Euclidean distance to nearest paved roads	Vehicule collisions constitute an important threat in northern Chile	Vargas, Bonacic & Moraga (2016)
Vegetation communities	Main vegetal communities described in the study region	Determinant role of local vegetation in habitat selection	Puig et al. (2008)
Protected areas	Protected areas along the Coquimbo and Atacama regions	Protected areas bring safety, stability and resources to the fauna	Geldmann et al. (2013) and Gray et al. (2016)

### Habitat suitability modelling and resistance surface

Habitat suitability was modelled based on a maximum entropy approach using MaxEnt version 3.3.3k (Phillips, Anderson & Schapire, 2006). MaxEnt is a machine-learning method that minimizes the relative entropy between the probability density at the presence sites and the probability density at background locations, the latter representing a random sample of the available environment (Elith et al., 2011). It is widely recognized as the most reliable approach in cases where only presence data are available (Phillips, Anderson & Schapire, 2006; Elith, Kearney & Phillips, 2010; Yackulic et al., 2013). The logistic output of MaxEnt provides a habitat suitability index (HSI) ranging from 0 to 1 (Phillips & Dudík, 2008; Anderson et al., 2016). We generated MaxEnt models using a bootstrap approach, where 70% of the occurrence data (i.e., 656 points) were used for training, while the remaining 30% (i.e., 281 points) were used to validate the model. A mask was applied to the study area, excluding non-continental areas and large areas with no historical occurrence data, in order to force MaxEnt to pick background information in areas within which the presence data were collected so that all the modelled data (presence and background) contained the same collection bias (Elith, Kearney & Phillips, 2010; Elith et al., 2011). We used the default number of background (or pseudo-absence) locations, 10,000.

To identify the best solution, MaxEnt uses a regularization multiplier and a set of features (i.e., transformations of the original predictor variables). Because the default settings can generate highly complex models (Kumar, Neven & Yee, 2014a; Kumar, Neven & Yee, 2014b), we first explored different combinations of features and various regularization multiplier values. For the selection of the features, we inspected the species responses (i.e., curves showing the probability of occurrence in relation to each predictor) obtained from various feature combinations. We opted for the linear and product features because their combined use resulted in simpler, more interpretable variable effects. The regularization multiplier is a smoothing parameter designed to reduce model overfitting and complexity (Radosavljevic & Anderson, 2014). To identify the optimal parameter value, we generated models with regularization multipliers varying from one to 20 with increments of one. Based on the Akaike Information Criteria corrected for small sample sizes (AICc), calculated using ENMTools (Warren, Glor & Turelli, 2008; Warren, Glor & Turelli, 2010; Warren & Seifert, 2011), optimal model performance was achieved using a regularization parameter of two. The collinearity of the variables was then analyzed by calculating Pearson correlations using the “raster” R-package (Hijmans et al., 2016). In cases where two variables were strongly correlated (|*r*| ≥ 0.75), we discarded the variable with the least ecological significance. The final set of environmental variables comprised elevation, distance to wetlands and rivers, vegetation communities, distance to protected areas, distance to urban settlements, and slope.

To construct the habitat suitability model, we ran 20 different bootstrap replicates and used the average results and area under the curve (AUC) scores. AUC scores are used to evaluate model performance, with values of one indicating a perfect fit of the presence data, and values close to 0.5 indicating that the model does not better predict the presence data than random background locations (Elith et al., 2011). The suitable habitat threshold was defined as the HSI value that maximized the sum of sensitivity (correct predictions of the occurrence) and specificity (correct predictions of the absence), as recommended by Liu, White & Newell (2013). To assess the performance of the model, we tested the significance of the extrinsic omission rate (i.e., the fraction of test localities falling outside the predicted suitable habitat) with a one-tailed binomial test (Phillips, Anderson & Schapire, 2006).

We derived the resistance surface from the habitat suitability scores by inverting and rescaling the HSI values into a continuous scale from one (low resistance/highly suitable for movement) to 100 (high resistance/low suitability for dispersal) using a linear scaling function available in ArcGIS 10.2.1 (ESRI, 2014). A barrier layer was then incorporated to generate the final dispersal cost map. We defined areas of intensive agriculture, towns and cities, mining extraction sites and large dams as impenetrable barriers to guanaco movement. Because vehicle collision is a leading cause of *L. guanicoe* mortality in the region (Vargas, Bonacic & Moraga, 2016), we considered unfenced highways as major barriers with a very low permeability, allocating them a very high resistance value (i.e., 95). Fencing potentially presents an absolute barrier to mammal movement (Van Langevelde, Van Dooremalen & Jaarsma, 2009) and decreases the survival probability of ungulates crossing highways, even in low traffic conditions (Harrington & Conover, 2006). We therefore allocated the maximum resistance value of 100 in fenced sections of the highway.

### Modelling landscape connectivity for *L. guanicoe*

We used Circuitscape 4.0 (McRae et al., 2008) to model connectivity and routes of dispersal across the landscape. Circuitscape, based on circuit theory, treats the landscape as a conductance surface, where each pixel represents a resistor with an assigned resistance (or, conversely, conductance) value. Pairwise electrical resistances between locations (McRae, 2006; McRae et al., 2008) are calculated by running a theoretical electrical current between each population pair, with one population being set as the current source and the other as the ground. Contrary to least cost resistance methods, Circuitscape does not assume that animals disperse according to previous knowledge of the surroundings, but is based on random walks (McRae, Shah & Edelman, 2016). It thus links populations through multiple pathways (McRae et al., 2008), such that connectivity between habitat patches increases according to the number of connected pathways, and the effective resistance between two populations is derived from the overall resistance across all pathways. To estimate effective resistance and densities, one ampere of current was injected to the current sources using the resistance surface derived from the habitat suitability model. A cumulative flow map based on all possible pairs of nodes was constructed displaying the amount of current flowing through each pixel according to the model. A map of maximum current densities between any pair of populations was also generated to identify areas that facilitate the most efficient movement between populations, and to identify pinch points, which correspond to areas where connectivity is most tenuous (McRae & Shah, 2009), and therefore essential for connectivity due to the lack of alternative pathways (i.e., McRae et al., 2008).

We used Linkage Mapper Connectivity Analysis Software (available at: http://www.circuitscape.org/linkagemapper) to build a network of least-cost corridors (McRae & Kavanagh, 2011). The resulting linkage network was then analyzed with the Centrality Mapper module to calculate current flow centrality (CFC) across the networks. CFC is a measure of the amount of dispersal passing through any given link or population as a function of its position in the network topology, thus allowing the contribution of each population and least-cost corridors to the linkage network to be assessed.

### Ethics statement

The capture and handling of guanacos for installation of tracking devices were performed according to the highest standards designed to ensure the safety of the animals. Prior approval was obtained from the Chilean authority for wildlife management (Servicio Agrícola y Ganadero—SAG; authorization No: 3346/2013 and 7899/2014), whose agents controlled all field manipulations of guanacos to ensure strict compliance with standards and regulations.

## Results

### Habitat suitability model generated by MaxEnt

**Figure 3:**
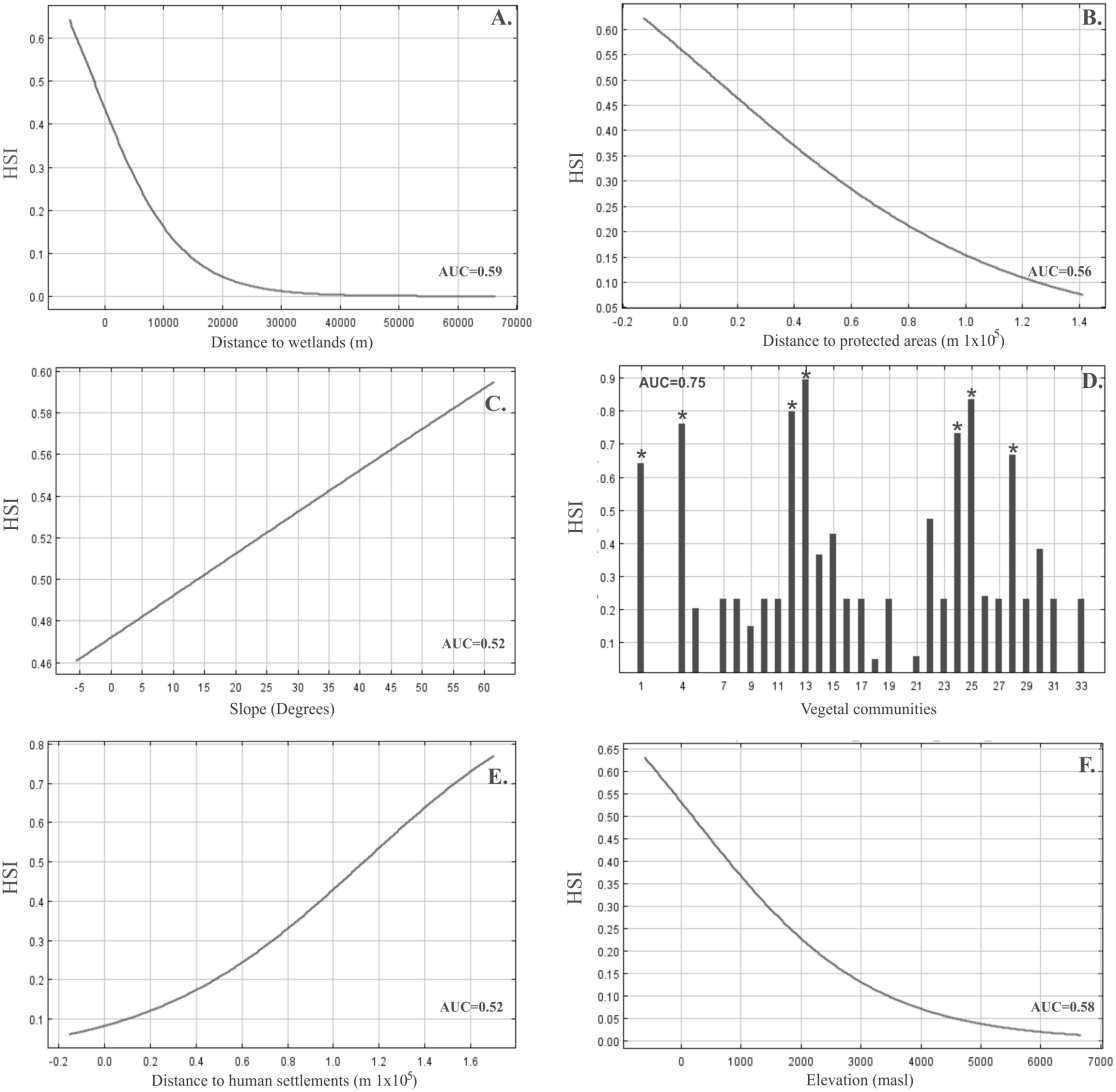
Response curves and predictive power of the environmental variables for the pruned habitat suitability model for *Lama guanicoe* generated by MaxEnt. Marginal response curves generated for each predictor are shown for continuous predictors (A) Distance to wetlands. (B) Distance to protected areas. (C) Slope. (E) Distance to human settlements. (F) Elevation. Single-variable response curve is shown for the categorical predictor. (D) Vegetal communities, each of them being described in Table S2. The predictive power of the variables, reported within the plot area in each case, is given by a Jackknife test of the variable importance using AUC on test data for each environmental variable retained in the MaxEnt model.

The final model of habitat suitability for *Lama guanicoe* across the study area performed better than random, with an average test AUC value of 0.87 (95% CI [0.84–0.88]; standard error: ±0.009). Extrinsic omission rate was 0.13 (*P* < 0.01), indicating that the variables of the pruned model contributed significantly to the habitat suitability predictions. Elevation, distance to wetlands, and vegetation communities were the most important predictors of habitat suitability, with a combined contribution of 87.7% to the final MaxEnt model (Table 2). The occurrence probability response of *L. guanicoe* to each predictor variable varied considerably, showing a strong negative association with distance to wetlands, distance to protected areas, and elevation (Figs. 3A, 3B and 3F), a positive association with scrubland vegetation communities (Andean Mediterranean sclerophyll forest, Andean Mediterranean underbrush, Andean tropical Mediterranean underbrush, Mediterranean pastureland, Mediterranean Coastal Desert Thicket, Mediterranean interior desert scrubland, Fig. 3D, Table S3), and a weak positive association with slope and distance to urban settlements (Figs. 3C and 3E). Maps of the six environmental variables, including guanaco occurrences, are provided in Fig. S1. Overall, our model predicted 29,173 km^2^ of suitable habitat heterogeneously distributed throughout the landscape, equivalent to approximately 25% of the total study area (Fig. S2A). Medium to high HSI values were found all along the Andes, forming a continuous stretch of suitable habitat from south to north of the study area up to the Nevado Tres Cruces National Park (Fig. S2B). By contrast, non-continuous patches of high HSI values surrounded by extensive zones of unsuitable habitat were predicted along the coast, particularly in the Limarí, Huasco, and Copiapó basins (Figs. S2A and S2B).

**Table 2:** Relative contribution of the environmental variables to the final habitat suitability model of *Lama guanicoe* in Chile’s Norte Chico.

Environmental variable	Contribution (%)
Vegetal communities	58.9
Elevation	15.6
Distance to wetlands and rivers	13.2
Distance to urbane settlements	7.2
Distance to protected areas	3.4
Slope	1.7

### Habitat resistance map of the study region for *L. guanicoe*

The resistance surface derived from the HSI scores showed a gradient of increasing resistance costs from south to north of the study area, with marked intermediate zones of high resistance costs within each river basin, being continued north of the Copiapó river basin (Fig. 2). Most of the areas of lower resistance cost were spatially coincident with high HSI value areas (see Fig. 2 and Fig. S2B), forming a contiguous expanse along high and mid elevation zones as far as the Copiapó river basin, and discontinuous patches along the coast, separated by all major transverse river basins except the Limarí. The Copiapó basin also represents the northern limit of the large predicted coastal areas with low habitat resistance, above which only a few discrete low resistance patches were identified (Fig. 2).

### Patterns of landscape connectivity for *L. guanicoe* across the study area

**Figure 4:**
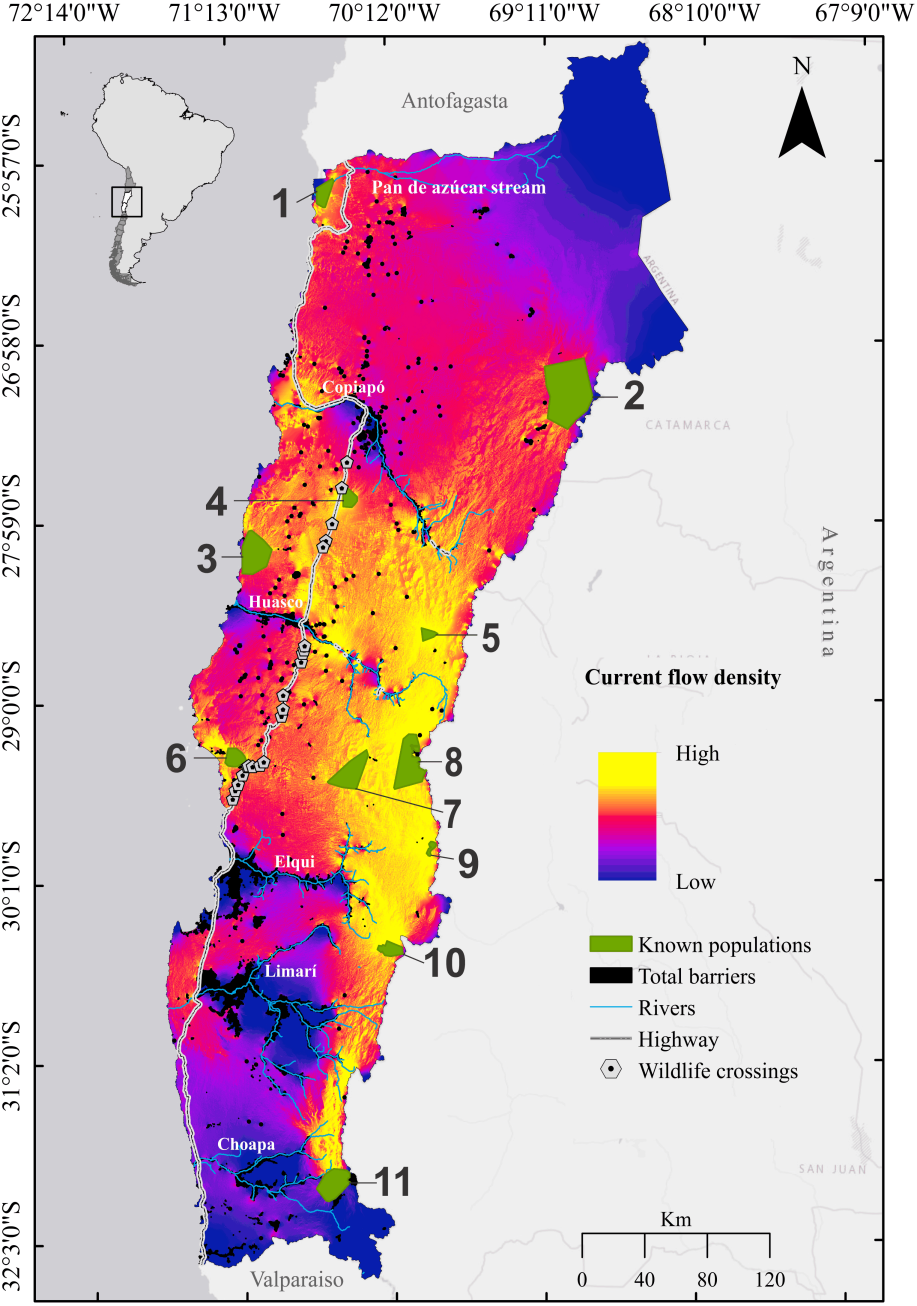
Cumulative current flow density map for the guanaco *Lama guanicoe* across Chile’s Norte Chico. Current flows represent passage probabilities calculated between all pairs of habitat patches corresponding to the guanaco populations by injecting 1 Ampere of current. Habitat patches corresponding to the guanaco populations are numbered as follow: **1**, Pan de Azúcar National Park; **2**, Nevado Tres Cruces National Park; **3**, Llanos de Challe National Park; **4**, Oso Negro sector; **5**, El Morro; **6**, Los Choros; **7**, Calvario stream; **8**, Tres Quebradas river; **9**, El Tambo stream; **10**, Estero Derecho nature sanctuary; **11**, Pelambres area.

**Figure 5:**
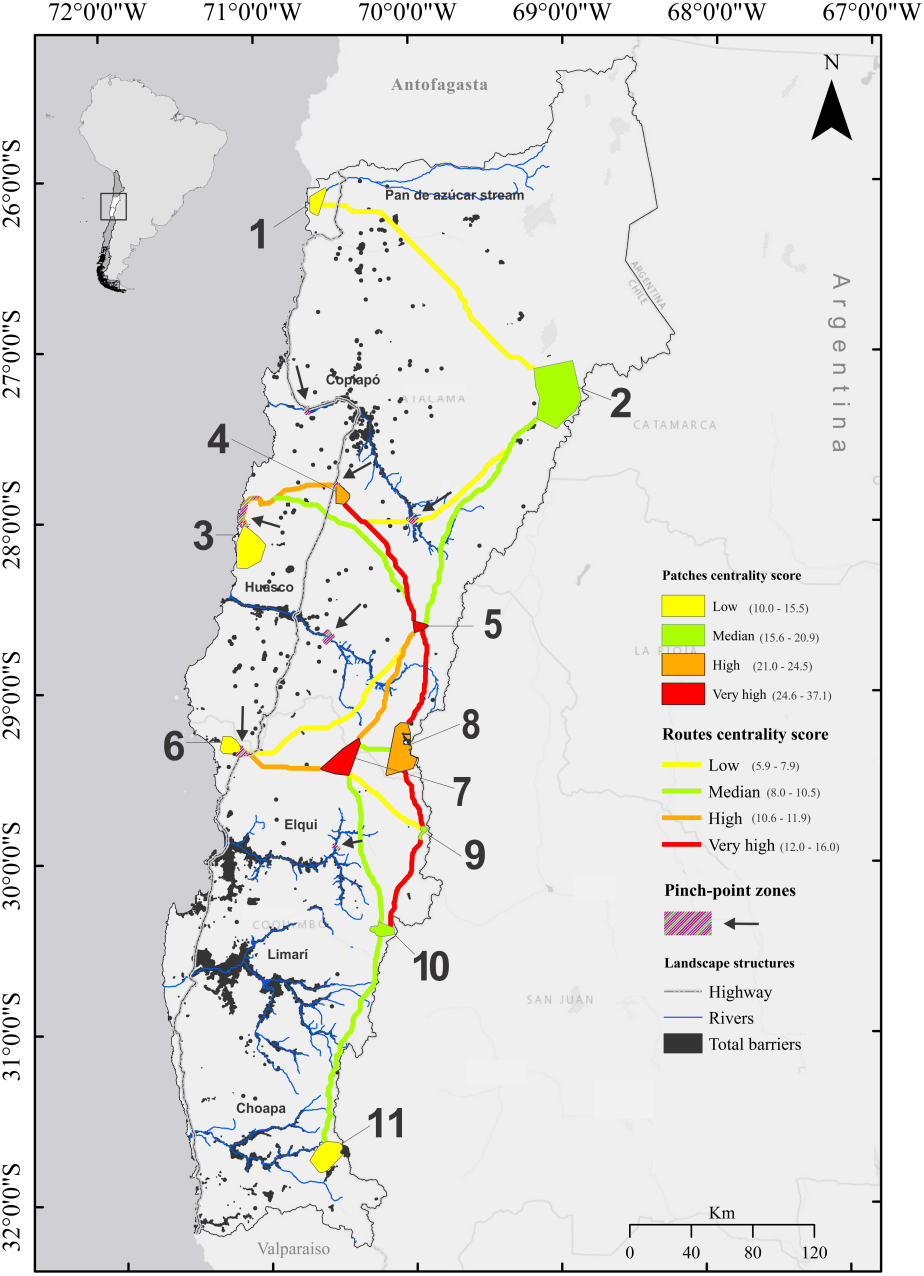
Linkage network map of *Lama guanicoe* populations in Chile’s Norte Chico, produced with the Linkage Mapper Connectivity Analysis software. Centrality score of patches and routes are represented on the map. Black arrows indicate locations identified as pinch points by the maximum cumulative current flow map generated by Circuistcape. Habitat patches corresponding to the guanaco populations are numbered as follow: **1**, Pan de Azúcar National Park; **2**, Nevado Tres Cruces National Park; **3**, Llanos de Challe National Park; **4**, Oso Negro sector; **5**, El Morro; **6**, Los Choros; **7**, Calvario stream; **8**, Tres Quebradas river; **9**, El Tambo stream; **10**, Estero Derecho nature sanctuary; **11**, Pelambres area.

The cumulative current density map based on all possible pairwise combinations between the 11 populations in the study area shows different current density patterns between coastal and mountainous areas. Similar to what was observed in the HSI and habitat resistance maps, highest cumulative current flow occurred within a wide corridor encompassing the mid and high elevation Andean sectors (28°00′02″–30°20′25″S and 69°45′0″–70°26′35″W), which harbor five guanaco populations (El Morro (5), Calvario (7), Tres Quebradas (8), El Tambo (9) and Estero Derecho (10); Fig. 4). Relatively high cumulative current flow was also found between the populations of Pelambres (11) and Estero Derecho (10) in the south of the study area. By contrast, current flows appeared discontinuous along the coast. Other areas with middle to high movement probabilities were revealed in the center of the study region between and along the transversal Elqui, Huasco and Copiapó valleys, as well as along the highway (Fig. 4). Overall, lowest current flows included the Pan de Azúcar (1) and Nevado Tres Cruces (2) national parks in the north and the Limarí and Choapa river basins in the south (Fig. 4). Seven pinch-points were identified by our maximum current flow model: one in the high Andes, two at mid altitudes, three at wildlife crossings located on the highway, and one in the coastal region (Fig. 5).

### Current flow centrality analysis between *L. guanicoe* populations across the study area

Our linkage map revealed a greater density of corridors connecting habitat patches above 600 masl between the Copiapó and Elqui river basins (Fig. 5). All three coastal populations were associated with relatively low centrality scores (Fig. 5). They connected to other populations by only one or two least resistance routes that crossed the highway, and which, in most cases (four out of five), harbored a pinch point (Fig. 5). No corridors directly linking coastal habitat patches were generated. Most pre-cordilleran and cordilleran habitat patches showed higher centrality scores and connected to other geographically close patches by at least two corridors. Only the northern and southernmost high Andean populations of Nevado Tres Cruces (2) and Pelambres (11), respectively, displayed low current flow centrality scores (Fig. 5), only receiving least cost paths with low or medium centrality scores.

## Discussion

### Habitat suitability of *Lama guanicoe* in Chile’s Norte Chico

In this study, we identified connectivity pathways for *L. guanicoe* in a region of Chile characterized by small and fragmented populations (Marín et al., 2013). To achieve this goal, we first developed a regional scale habitat suitability model. Consistent with González et al. (2013), we identified areas of suitable habitat along both the coastline as well as the Andes. Overall, predicted habitat suitability comprises an area of 29,173 km^2^, which slightly exceeds the prediction of González et al. (2013) for the same region (i.e., 23,481 km^2^). Differences in the predictor variables, the definition of the habitat suitability threshold or resolution of the models may explain the observed discrepancy. Because González et al. (2013) aimed to evaluate habitat suitability across the entire *L. guanicoe* distribution range in Chile, they used data layers of a much lower resolution (3 × 3 km) than ours (90 × 90 m). Models built over large areas are expected to have weak local predictive power due to regional niche variation (Osborne & Suárez-Seoane, 2002; Murphy & Lovett-Doust, 2007), and higher resolution models are therefore better suited for regional scale applications (Carroll, McRae & Brookes, 2012), as was the case here.

We found that resource factors most strongly influenced *L. guanicoe* distribution in Chile’s Norte Chico, followed by elevation and then disturbance factors. Vegetation and distance to water resources accounted for 72% of the predictive ability of the Maxent model; constraining *L. guanicoe*’s presence to seven of the 33 vegetation communities of the study area (Luebert & Pliscoff, 2006), and to areas located less than 5 km from water resources. The seven vegetation communities included at least one plant species foraged by *L. guanicoe* (Table S3). The importance of the resource factors identified in this study is consistent with Lucherini et al. (2000)’s habitat use study in a high Andean ecosystem of North-Eastern Argentina, which showed that free-ranging guanacos most often occurred in vegetation-rich areas close to streams. Overall, these results suggest that forage and water availability are key drivers of guanaco distribution patterns, at least in environments where resources are limited and heterogeneously distributed. In the study area, these factors resulted in a heterogeneous distribution of habitat suitable for *L. guanicoe*. The largest sector was predicted in the foothills of the mid and high elevation areas (2,000–4,500 masl), where four of the influential vegetation communities (Andean Mediterranean sclerophyll forest of *K. angustifolia* and *G. trinervis,* Mediterranean pastureland of *N. spathulatus* and *M. spathulata*, Andean tropical Mediterranean underbrush *A. subterranea* and *A. echinus*, Andean Mediterranean underbrush *Laretia acaulis* and *Berberis empetrifolia*) as well as numerous Andean wetlands occur (Squeo, Arancio & Gutiérrez, 2001; Squeo et al., 2006; Squeo et al., 2008). High Andean wetlands may not only provide water supply for *L. guanicoe,* but also fulfill various other needs such as food and shelter (Torres, 1992). The other three influential vegetation communities (Mediterranean Coastal Desert Thicket *O. gigantean* and *E. breviflora,* Mediterranean Coastal Desert Thicket *H. stenophyllum*, Mediterranean interior desert scrubland *H. stenophyllum* and *F. thurifera*) are constrained to coastal areas, and in a section excluding the southernmost and northernmost regions. In the coastal areas, water resources are sparse and scattered, resulting in large stretches of unsuitable habitat along the coast.

Elevation accounted for 16% of the model’s predictive ability, with occurrence probabilities gradually declining with increases in elevation. However, even at the highest elevations, the HSI values did not fall below the habitat suitability threshold. This finding is consistent with literature reports of physiological and physical adaptations of guanacos to high altitude (Wilson, 1989; Starck & Wang, 2005). Compared to resource factors and elevation, disturbance factors only moderately influenced *L. guanicoe* distribution. Distance to urban settlements and distance to protected areas accounted for a combined 10.6% of the predictive ability of the model; as anticipated, proximity to protected areas exerted a positive effect on occurrence probability, while proximity to human settlements had the opposite effect. While none of the distance to human settlements was associated with HSI corresponding to unsuitable habitat, the fact that adverse effects were detected suggests that the current growth in urbanization in the region (INE, 2012) may become a serious threat for *L. guanicoe* in the near future.

### Landscape connectivity and conservation priorities

The proportion of predicted suitable habitat (i.e., 25%) fell below the threshold at which patch isolation increases the risk of extinction in bird and mammal populations (Andrén, 1994; Betts et al., 2006), indicating that population connectivity is an issue of concern for *L. guanicoe* in Chile’s Norte Chico. In this context, our study contributes pertinent knowledge. Clear connectivity patterns were identified, including both connectivity corridors and hotspots, as well as areas of low movement probability and functionally isolated populations. The area most permeable to *L. guanicoe* movement was predicted in the Andes, in a sector spanning about 2/3 of the latitudinal extent of the study area. This corridor enables movement between all the high Andean populations, of which five (Estero Derecho, El Tambo, Calvario, Tres-quebradas and El Morro) in particular demonstrate high probability of inter-population movement, being crossed by multiple pathways. Only a single pinch point was detected in this area, located upstream of the Elqui river, but did not affect connectivity since it was not located on any connectivity pathway. Altogether, these results suggest a relatively high resilience of the population network in the pre-cordilleran and cordilleran regions, which could further benefit from transboundary movements with the Argentinean populations.

Population connectivity nevertheless remains an issue of concern in the Andean region, which is facing multiple threats due to mining (Squeo et al., 2006, Table S4) including habitat loss and water contamination, exacerbated by the high density of mining concessions located close to important water resources such as high-altitude wetlands (Troncoso et al., 2017). In the absence of adequate regulation, mining therefore has the potential to significantly increase habitat fragmentation in this sector. Environmental impact assessments and mitigation programs should thus consider population connectivity in future baseline studies, and particularly for those populations that may be at higher extinction risk, such as El Tambo and Nevado Tres Cruces National Park, which may be less resilient to local threats due to small population size (i.e., *N* < 80, Table S4), and Nevado Tres Cruces National Park, which displayed a low centrality score. Because our estimates did not consider populations outside the study area, some neighborhood populations of the Andean sites might have been omitted, which may have resulted in an underestimate of the centrality scores in the cordilleran region. Further analyses, such as population genetic studies, are needed to confirm the isolation status of the Nevado Tres Cruces National Park.

Connectivity patterns along the coast contrasted strongly with those observed in the Andes. The coastal landscape was essentially dominated by low to medium current areas. Low current areas reflect either barriers to movement or very large corridors (Cushman, Chase & Griffin, 2010). In the present case, they occurred in high-resistance areas (i.e., low quality habitat), coinciding with urban areas or areas of intensive agriculture (Novoa & López, 2001). As a result, no connectivity was detected between coastal populations themselves, only to populations at higher altitudes. The actual effectiveness of these connections was unclear, however. Indeed, they implied crossing the four-lane highway that extends vertically across the study area. Fenced highways increase animal mortalities due to vehicle collisions and animals becoming trapped in the barbed wire (Vanak, Thaker & Slotow, 2010). Besides, two pinch points along the highway were coincident with locations of wildlife underpasses, located in the pathways linking Llanos del Challe to Oso Negro and Los Choros to El Cavario. To date, it is unknown if guanacos utilize these structures effectively. This should be a topic of future research, particularly since evidence suggests that ungulates tend to demonstrate a preference for utilizing overpasses rather than underpasses (Simpson et al., 2016). Overall, our results suggest that the coastal populations may be functionally isolated, a situation that would endanger their long-term persistence. This threat could be further compounded if anthropogenic activities like mining were developed in the mid elevation sector, which could severely affect the few remaining connectivity links of the coastal populations. Actions to protect or restore connectivity might thus be crucial for the conservation of the remnant coastal populations of *L. guanicoe*.

Other areas that should be prioritized are those playing a key role in the connectivity network. Our model recognized El Morro and Calvario patches as the most important habitat patches for overall connectivity, facilitating individuals’ dispersal between several pairs of populations. If these resource patches were to be lost, it would result in considerable increases in the distance and/or transit times between populations (Carroll, McRae & Brookes, 2012). Future regional planning should consider maintaining their integrity for the long-term persistence of this emblematic species in this region of Chile.

## Conclusions

The identification of biological corridors, defined as areas of natural habitat that allow species dispersal processes essential for their persistence in a landscape, is of prime importance for the conservation of endangered species, and also has implications for the maintenance of important biological patterns and processes at large regional scales (Chetkiewicz, Clair & Boyce, 2006; Rouget et al., 2006). In this study, we used a resistance-surface-based connectivity modelling approach to investigate functional connectivity of *L. guanicoe* in Chile’s Norte Chico. To appraise the actual pertinence of our results in terms of dispersal, future studies contrasting our connectivity model predictions against gene flow would be needed (Baguette et al., 2013). Yet our study suggests that functional connectivity is an issue of concern for *L. guanicoe* in Chile’s Norte Chico. Indeed, we found that isolation may jeopardize the viability of the three coastal populations, which are the last remaining in Chile. Very few of the connectivity pathways may in fact facilitate access to these populations, and the effectiveness of these routes needs to be investigated, since their functionality appears to be wholly dependent on wildlife crossing structures that may or may not be appropriate for *L. guanicoe*. Our results were rather comforting for most Andean populations, for which we predicted high connectivity levels; and two populations in particular were found to play a central role in the connectivity network. Collectively, these results indicate that future conservation and management plans involving *L. guanicoe* in the region should adopt a landscape strategy designed to conserve functional connectivity between coastal and Andean populations, as well as protect habitat patches that likely function as stepping stones within the connectivity network.

##  Supplemental Information

10.7717/peerj.4429/supp-1 Table S1 Occurrence data used for habitat suitability modelling of *Lama guanicoe* in the Chile’s Norte Chico

10.7717/peerj.4429/supp-2 Table S2 Vegetation communities used for habitat suitability modelling of *Lama guanicoe* in Chile’s Norte Chico Source: (Luebert & Pliscoff, 2006).

10.7717/peerj.4429/supp-3 Table S3 Food items listed according to species and vegetation communities contributing strongly to *Lama guanicoe* distribution in the study area

10.7717/peerj.4429/supp-4 Table S4 Size, protection status, current flow centrality and threats faced by *Lama guanicoe* populations in Chile’s Norte Chico

10.7717/peerj.4429/supp-5 Figure S1 Maps of the six environmental variables including guanaco presence data used for habitat suitability modelling in Chile’s Norte Chico (see Methodology).

10.7717/peerj.4429/supp-6 Figure S2 Potential suitable habitat (suitable/unsuitable) (A) and Habitat suitability index (B) maps of *Lama guanicoe* across Chile’s Norte Chico The suitable habitat threshold was defined as the habitat suitability index value that maximized the sum of sensitivity and specificity (see Methodology). Habitat patches corresponding to the guanaco populations are represented in Figure part B: **1**, Pan de Azúcar National Park; **2**, Nevado Tres Cruces National Park; **3**, Llanos de Challe National Park; **4**, Oso Negro sector; **5**, El Morro; **6**, Los Choros; **7**, Calvario stream; **8**, Tres Quebradas river; **9**, El Tambo stream; **10**, Estero Derecho nature sanctuary; **11**, Pelambres area.

## Additional Information and Declarations

### Data Availability

The following information was supplied regarding data availability: Espinosa Herazo, Mara Inés; Gouin, Nicolas; Squeo, Francisco Antonio; López, David; Bertin, Angéline (2018): Landscape connectivity among remnant populations of guanaco (Lama guanicoe, Müller, 1776) in an arid region of Chile impacted by global change. figshare. https://dx.doi.org/10.6084/m9.figshare.5378233.v3.
