# Supplementary material for: Landscape connectivity among remnant populations of guanaco (Lama guanicoe Müller, 1776) in an arid region of Chile impacted by global change
Source: PeerJ. 2018 Mar 2;6:e4429. doi: 10.7717/peerj.4429 (PMC5836568; doi:10.7717/peerj.4429)
Supplement: Table S1 [file peerj-06-4429-s001.docx]

Supplementary Table S1

Occurrence data used for habitat suitability modelling of *Lama guanicoe* in the Chile’s Norte Chico.

| **Species** | **Latitude** | **Longitude** | **Source** | **Sector** | **Year** |
| --- | --- | --- | --- | --- | --- |
| Guanaco | 6739143.3 | 342706 | Feces - Sighting | Calvario stream | 2012 - 2014 |
| Guanaco | 6738643.3 | 343206 | Feces - Sighting | Calvario stream | 2012 - 2014 |
| Guanaco | 6738143.3 | 343706 | Feces - Sighting | Calvario stream | 2012 - 2014 |
| Guanaco | 6738643.3 | 343706 | Feces - Sighting | Calvario stream | 2012 - 2014 |
| Guanaco | 6738143.3 | 347206 | Feces - Sighting | Calvario stream | 2012 - 2014 |
| Guanaco | 6737143.3 | 349206 | Feces - Sighting | Calvario stream | 2012 - 2014 |
| Guanaco | 6736643.3 | 350206 | Feces - Sighting | Calvario stream | 2012 - 2014 |
| Guanaco | 6736643.3 | 351206 | Feces - Sighting | Calvario stream | 2012 - 2014 |
| Guanaco | 6736643.3 | 351706 | Feces - Sighting | Calvario stream | 2012 - 2014 |
| Guanaco | 6735643.3 | 352206 | Feces - Sighting | Calvario stream | 2012 - 2014 |
| Guanaco | 6736143.3 | 352206 | Feces - Sighting | Calvario stream | 2012 - 2014 |
| Guanaco | 6736643.3 | 352706 | Feces - Sighting | Calvario stream | 2012 - 2014 |
| Guanaco | 6737643.3 | 353206 | Feces - Sighting | Calvario stream | 2012 - 2014 |
| Guanaco | 6738643.3 | 353206 | Feces - Sighting | Calvario stream | 2012 - 2014 |
| Guanaco | 6744643.3 | 353206 | Feces - Sighting | Calvario stream | 2012 - 2014 |
| Guanaco | 6741143.3 | 353706 | Feces - Sighting | Calvario stream | 2012 - 2014 |
| Guanaco | 6743143.3 | 353706 | Feces - Sighting | Calvario stream | 2012 - 2014 |
| Guanaco | 6741143.3 | 354206 | Feces - Sighting | Calvario stream | 2012 - 2014 |
| Guanaco | 6742143.3 | 354206 | Feces - Sighting | Calvario stream | 2012 - 2014 |
| Guanaco | 6742643.3 | 354206 | Feces - Sighting | Calvario stream | 2012 - 2014 |
| Guanaco | 6743143.3 | 354206 | Feces - Sighting | Calvario stream | 2012 - 2014 |
| Guanaco | 6741143.3 | 354706 | Feces - Sighting | Calvario stream | 2012 - 2014 |
| Guanaco | 6743143.3 | 354706 | Feces - Sighting | Calvario stream | 2012 - 2014 |
| Guanaco | 6743643.3 | 354706 | Feces - Sighting | Calvario stream | 2012 - 2014 |
| Guanaco | 6739643.3 | 355206 | Feces - Sighting | Calvario stream | 2012 - 2014 |
| Guanaco | 6743643.3 | 355706 | Feces - Sighting | Calvario stream | 2012 - 2014 |
| Guanaco | 6744143.3 | 355706 | Feces - Sighting | Calvario stream | 2012 - 2014 |
| Guanaco | 6744643.3 | 356206 | Feces - Sighting | Calvario stream | 2012 - 2014 |
| Guanaco | 6745143.3 | 356206 | Feces - Sighting | Calvario stream | 2012 - 2014 |
| Guanaco | 6742643.3 | 356706 | Feces - Sighting | Calvario stream | 2012 - 2014 |
| Guanaco | 6743143.3 | 356706 | Feces - Sighting | Calvario stream | 2012 - 2014 |
| Guanaco | 6745643.3 | 356706 | Feces - Sighting | Calvario stream | 2012 - 2014 |
| Guanaco | 6747143.3 | 356706 | Feces - Sighting | Calvario stream | 2012 - 2014 |
| Guanaco | 6748143.3 | 357206 | Feces - Sighting | Calvario stream | 2012 - 2014 |
| Guanaco | 6750143.3 | 357206 | Feces - Sighting | Calvario stream | 2012 - 2014 |
| Guanaco | 6750643.3 | 357206 | Feces - Sighting | Calvario stream | 2012 - 2014 |
| Guanaco | 6751143.3 | 357206 | Feces - Sighting | Calvario stream | 2012 - 2014 |
| Guanaco | 6751643.3 | 357206 | Feces - Sighting | Calvario stream | 2012 - 2014 |
| Guanaco | 6752143.3 | 357206 | Feces - Sighting | Calvario stream | 2012 - 2014 |
| Guanaco | 6736143.3 | 357706 | Feces - Sighting | Calvario stream | 2012 - 2014 |
| Guanaco | 6747143.3 | 357706 | Feces - Sighting | Calvario stream | 2012 - 2014 |
| Guanaco | 6749143.3 | 357706 | Feces - Sighting | Calvario stream | 2012 - 2014 |
| Guanaco | 6749643.3 | 357706 | Feces - Sighting | Calvario stream | 2012 - 2014 |
| Guanaco | 6750143.3 | 357706 | Feces - Sighting | Calvario stream | 2012 - 2014 |
| Guanaco | 6750643.3 | 357706 | Feces - Sighting | Calvario stream | 2012 - 2014 |
| Guanaco | 6751643.3 | 357706 | Feces - Sighting | Calvario stream | 2012 - 2014 |
| Guanaco | 6752143.3 | 357706 | Feces - Sighting | Calvario stream | 2012 - 2014 |
| Guanaco | 6749643.3 | 358206 | Feces - Sighting | Calvario stream | 2012 - 2014 |
| Guanaco | 6750143.3 | 358206 | Feces - Sighting | Calvario stream | 2012 - 2014 |
| Guanaco | 6735643.3 | 358706 | Feces - Sighting | Calvario stream | 2012 - 2014 |
| Guanaco | 6749143.3 | 358706 | Feces - Sighting | Calvario stream | 2012 - 2014 |
| Guanaco | 6749643.3 | 358706 | Feces - Sighting | Calvario stream | 2012 - 2014 |
| Guanaco | 6750143.3 | 358706 | Feces - Sighting | Calvario stream | 2012 - 2014 |
| Guanaco | 6751643.3 | 359206 | Feces - Sighting | Calvario stream | 2012 - 2014 |
| Guanaco | 6735643.3 | 359706 | Feces - Sighting | Calvario stream | 2012 - 2014 |
| Guanaco | 6735643.3 | 360206 | Feces - Sighting | Calvario stream | 2012 - 2014 |
| Guanaco | 6752143.3 | 360706 | Feces - Sighting | Calvario stream | 2012 - 2014 |
| Guanaco | 6752643.3 | 360706 | Feces - Sighting | Calvario stream | 2012 - 2014 |
| Guanaco | 6734643.3 | 361206 | Feces - Sighting | Calvario stream | 2012 - 2014 |
| Guanaco | 6735143.3 | 361206 | Feces - Sighting | Calvario stream | 2012 - 2014 |
| Guanaco | 6735643.3 | 361206 | Feces - Sighting | Calvario stream | 2012 - 2014 |
| Guanaco | 6752143.3 | 361206 | Feces - Sighting | Calvario stream | 2012 - 2014 |
| Guanaco | 6753143.3 | 361206 | Feces - Sighting | Calvario stream | 2012 - 2014 |
| Guanaco | 6753643.3 | 361206 | Feces - Sighting | Calvario stream | 2012 - 2014 |
| Guanaco | 6752643.3 | 361706 | Feces - Sighting | Calvario stream | 2012 - 2014 |
| Guanaco | 6753143.3 | 361706 | Feces - Sighting | Calvario stream | 2012 - 2014 |
| Guanaco | 6754143.3 | 362706 | Feces - Sighting | Calvario stream | 2012 - 2014 |
| Guanaco | 6754143.3 | 363206 | Feces - Sighting | Calvario stream | 2012 - 2014 |
| Guanaco | 6754143.3 | 364206 | Feces - Sighting | Calvario stream | 2012 - 2014 |
| Guanaco | 6754143.3 | 365206 | Feces - Sighting | Calvario stream | 2012 - 2014 |
| Guanaco | 6755143.3 | 365706 | Feces - Sighting | Calvario stream | 2012 - 2014 |
| Guanaco | 6755643.3 | 366206 | Feces - Sighting | Calvario stream | 2012 - 2014 |
| Guanaco | 6756643.3 | 366706 | Feces - Sighting | Calvario stream | 2012 - 2014 |
| Guanaco | 6756143.3 | 367206 | Feces - Sighting | Calvario stream | 2012 - 2014 |
| Guanaco | 6756643.3 | 367206 | Feces - Sighting | Calvario stream | 2012 - 2014 |
| Guanaco | 6756143.3 | 367706 | Feces - Sighting | Calvario stream | 2012 - 2014 |
| Guanaco | 6756643.3 | 367706 | Feces - Sighting | Calvario stream | 2012 - 2014 |
| Guanaco | 6759143.3 | 367706 | Feces - Sighting | Calvario stream | 2012 - 2014 |
| Guanaco | 6755643.3 | 368706 | Feces - Sighting | Calvario stream | 2012 - 2014 |
| Guanaco | 6931743.3 | 302106 | González et al. (2013) | Coastal sector | 2000-2011 |
| Guanaco | 6930343.3 | 303506 | González et al. (2013) | Coastal sector | 2000-2011 |
| Guanaco | 6931043.3 | 303506 | González et al. (2013) | Coastal sector | 2000-2011 |
| Guanaco | 6930343.3 | 304206 | González et al. (2013) | Coastal sector | 2000-2011 |
| Guanaco | 6928243.3 | 304906 | González et al. (2013) | Coastal sector | 2000-2011 |
| Guanaco | 6930343.3 | 304906 | González et al. (2013) | Coastal sector | 2000-2011 |
| Guanaco | 6951343.3 | 335006 | González et al. (2013) | Coastal sector | 2000-2011 |
| Guanaco | 6940843.3 | 338506 | Feces - Sighting | Coastal sector | 2012 - 2014 |
| Guanaco | 6942943.3 | 338506 | Feces - Sighting | Coastal sector | 2012 - 2014 |
| Guanaco | 6944343.3 | 338506 | Feces - Sighting | Coastal sector | 2012 - 2014 |
| Guanaco | 6947143.3 | 338506 | Feces - Sighting | Coastal sector | 2012 - 2014 |
| Guanaco | 6939443.3 | 339206 | Feces - Sighting | Coastal sector | 2012 - 2014 |
| Guanaco | 6940143.3 | 339206 | Feces - Sighting | Coastal sector | 2012 - 2014 |
| Guanaco | 6942943.3 | 339206 | Feces - Sighting | Coastal sector | 2012 - 2014 |
| Guanaco | 6945043.3 | 339206 | Feces - Sighting | Coastal sector | 2012 - 2014 |
| Guanaco | 6946443.3 | 339206 | Feces - Sighting | Coastal sector | 2012 - 2014 |
| Guanaco | 6948543.3 | 339206 | Feces - Sighting | Coastal sector | 2012 - 2014 |
| Guanaco | 6947843.3 | 339906 | Feces - Sighting | Coastal sector | 2012 - 2014 |
| Guanaco | 6949943.3 | 339906 | Feces - Sighting | Coastal sector | 2012 - 2014 |
| Guanaco | 6951343.3 | 339906 | Feces - Sighting | Coastal sector | 2012 - 2014 |
| Guanaco | 6952043.3 | 340606 | Feces - Sighting | Coastal sector | 2012 - 2014 |
| Guanaco | 6956943.3 | 340606 | Feces - Sighting | Coastal sector | 2012 - 2014 |
| Guanaco | 6951343.3 | 341306 | González et al. (2013) | Coastal sector | 2000-2011 |
| Guanaco | 6954843.3 | 341306 | Feces - Sighting | Coastal sector | 2012 - 2014 |
| Guanaco | 6956243.3 | 341306 | Feces - Sighting | Coastal sector | 2012 - 2014 |
| Guanaco | 6930343.3 | 342006 | Feces - Sighting | Coastal sector | 2012 - 2014 |
| Guanaco | 6932443.3 | 342006 | Feces - Sighting | Coastal sector | 2012 - 2014 |
| Guanaco | 6953443.3 | 342006 | Feces - Sighting | Coastal sector | 2012 - 2014 |
| Guanaco | 6954843.3 | 342006 | Feces - Sighting | Coastal sector | 2012 - 2014 |
| Guanaco | 6958343.3 | 342006 | Feces - Sighting | Coastal sector | 2012 - 2014 |
| Guanaco | 6928943.3 | 342706 | Feces - Sighting | Coastal sector | 2012 - 2014 |
| Guanaco | 6929643.3 | 342706 | Feces - Sighting | Coastal sector | 2012 - 2014 |
| Guanaco | 6925443.3 | 343406 | Feces - Sighting | Coastal sector | 2012 - 2014 |
| Guanaco | 6926843.3 | 343406 | Feces - Sighting | Coastal sector | 2012 - 2014 |
| Guanaco | 6927543.3 | 343406 | Feces - Sighting | Coastal sector | 2012 - 2014 |
| Guanaco | 6926843.3 | 348306 | Researchers contribution | Coastal sector | 2008-2012 |
| Guanaco | 6926843.3 | 349006 | Researchers contribution | Coastal sector | 2008-2012 |
| Guanaco | 6928243.3 | 349006 | Researchers contribution | Coastal sector | 2008-2012 |
| Guanaco | 6928943.3 | 349706 | Researchers contribution | Coastal sector | 2008-2012 |
| Guanaco | 6929643.3 | 349706 | Researchers contribution | Coastal sector | 2008-2012 |
| Guanaco | 6931743.3 | 352506 | Researchers contribution | Coastal sector | 2008-2012 |
| Guanaco | 6932443.3 | 352506 | Researchers contribution | Coastal sector | 2008-2012 |
| Guanaco | 6931043.3 | 353206 | Researchers contribution | Coastal sector | 2008-2012 |
| Guanaco | 6795643.3 | 264206 | Researchers contribution | Coastal sector | 2008-2012 |
| Guanaco | 6805143.3 | 265206 | Bonacic et al. (2014) | Coastal sector | 2014 |
| Guanaco | 6807143.3 | 270706 | Bonacic et al. (2014) | Coastal sector | 2014 |
| Guanaco | 6794143.3 | 296206 | Researchers contribution | Coastal sector | 2008-2012 |
| Guanaco | 6997143.3 | 308206 | González et al. (2013) | Coastal sector | 2000-2011 |
| Guanaco | 6922143.3 | 310206 | González et al. (2013) | Coastal sector | 2000-2011 |
| Guanaco | 6923143.3 | 310206 | González et al. (2013) | Coastal sector | 2000-2011 |
| Guanaco | 6922643.3 | 310706 | González et al. (2013) | Coastal sector | 2000-2011 |
| Guanaco | 6927643.3 | 315206 | González et al. (2013) | Coastal sector | 2000-2011 |
| Guanaco | 6927143.3 | 317206 | González et al. (2013) | Coastal sector | 2000-2011 |
| Guanaco | 6926143.3 | 319206 | González et al. (2013) | Coastal sector | 2000-2011 |
| Guanaco | 6946643.3 | 321206 | González et al. (2013) | Coastal sector | 2000-2011 |
| Guanaco | 6924643.3 | 322706 | González et al. (2013) | Coastal sector | 2000-2011 |
| Guanaco | 6947643.3 | 323206 | González et al. (2013) | Coastal sector | 2000-2011 |
| Guanaco | 6941643.3 | 323706 | González et al. (2013) | Coastal sector | 2000-2011 |
| Guanaco | 6921643.3 | 326706 | González et al. (2013) | Coastal sector | 2000-2011 |
| Guanaco | 6921143.3 | 327206 | González et al. (2013) | Coastal sector | 2000-2011 |
| Guanaco | 6919643.3 | 329206 | González et al. (2013) | Coastal sector | 2000-2011 |
| Guanaco | 6917143.3 | 332706 | González et al. (2013) | Coastal sector | 2000-2011 |
| Guanaco | 6947643.3 | 348706 | González et al. (2013) | Coastal sector | 2000-2011 |
| Guanaco | 6948643.3 | 357206 | González et al. (2013) | Coastal sector | 2000-2011 |
| Guanaco | 6949143.3 | 358706 | González et al. (2013) | Coastal sector | 2000-2011 |
| Guanaco | 6858143.3 | 405706 | Researchers contribution | Cordillera | 2008-2012 |
| Guanaco | 6896643.3 | 411206 | Bonacic et al. (2014) | Cordillera | 2014 |
| Guanaco | 6666143.3 | 411706 | Researchers contribution | Cordillera | 2008-2012 |
| Guanaco | 6896143.3 | 411706 | Bonacic et al. (2014) | Cordillera | 2014 |
| Guanaco | 6665643.3 | 413206 | Researchers contribution | Cordillera | 2008-2012 |
| Guanaco | 6895643.3 | 413706 | Bonacic et al. (2014) | Cordillera | 2014 |
| Guanaco | 6896143.3 | 413706 | Bonacic et al. (2014) | Cordillera | 2014 |
| Guanaco | 6666143.3 | 414206 | Researchers contribution | Cordillera | 2008-2012 |
| Guanaco | 6895643.3 | 414706 | Bonacic et al. (2014) | Cordillera | 2014 |
| Guanaco | 6894643.3 | 415706 | Bonacic et al. (2014) | Cordillera | 2014 |
| Guanaco | 6895143.3 | 417706 | Bonacic et al. (2014) | Cordillera | 2014 |
| Guanaco | 6894143.3 | 419706 | Bonacic et al. (2014) | Cordillera | 2014 |
| Guanaco | 6893643.3 | 421206 | Bonacic et al. (2014) | Cordillera | 2014 |
| Guanaco | 6897143.3 | 422706 | Bonacic et al. (2014) | Cordillera | 2014 |
| Guanaco | 6894643.3 | 423206 | Bonacic et al. (2014) | Cordillera | 2014 |
| Guanaco | 6897643.3 | 424706 | Bonacic et al. (2014) | Cordillera | 2014 |
| Guanaco | 6935143.3 | 426706 | Feces - Sighting | Cordillera | 2012 - 2014 |
| Guanaco | 6889143.3 | 429206 | Bonacic et al. (2014) | Cordillera | 2014 |
| Guanaco | 6932143.3 | 430706 | Feces - Sighting | Cordillera | 2012 - 2014 |
| Guanaco | 6887143.3 | 432706 | Bonacic et al. (2014) | Cordillera | 2014 |
| Guanaco | 6884643.3 | 435206 | Bonacic et al. (2014) | Cordillera | 2014 |
| Guanaco | 6886643.3 | 436706 | González et al. (2013) | Cordillera | 2000-2011 |
| Guanaco | 6881143.3 | 438206 | Bonacic et al. (2014) | Cordillera | 2014 |
| Guanaco | 6950643.3 | 451706 | González et al. (2013) | Cordillera | 2000-2011 |
| Guanaco | 6998643.3 | 462206 | González et al. (2013) | Cordillera | 2000-2011 |
| Guanaco | 6996643.3 | 465706 | González et al. (2013) | Cordillera | 2000-2011 |
| Guanaco | 6910643.3 | 467206 | Feces - Sighting | Cordillera | 2012 - 2014 |
| Guanaco | 6910643.3 | 467706 | Feces - Sighting | Cordillera | 2012 - 2014 |
| Guanaco | 6946643.3 | 471706 | González et al. (2013) | Cordillera | 2000-2011 |
| Guanaco | 6957643.3 | 472206 | González et al. (2013) | Cordillera | 2000-2011 |
| Guanaco | 7077143.3 | 472206 | Bonacic et al. (2014) | Cordillera | 2014 |
| Guanaco | 6958643.3 | 473206 | González et al. (2013) | Cordillera | 2000-2011 |
| Guanaco | 6919143.3 | 473706 | Feces - Sighting | Cordillera | 2012 - 2014 |
| Guanaco | 6921143.3 | 474206 | Feces - Sighting | Cordillera | 2012 - 2014 |
| Guanaco | 6961143.3 | 474206 | González et al. (2013) | Cordillera | 2000-2011 |
| Guanaco | 7093143.3 | 474206 | González et al. (2013) | Cordillera | 2000-2011 |
| Guanaco | 7097143.3 | 474206 | González et al. (2013) | Cordillera | 2000-2011 |
| Guanaco | 6960143.3 | 474706 | González et al. (2013) | Cordillera | 2000-2011 |
| Guanaco | 7086643.3 | 474706 | Bonacic et al. (2014) | Cordillera | 2014 |
| Guanaco | 7087143.3 | 474706 | González et al. (2013) | Cordillera | 2000-2011 |
| Guanaco | 7087643.3 | 474706 | González et al. (2013) | Cordillera | 2000-2011 |
| Guanaco | 7088143.3 | 474706 | González et al. (2013) | Cordillera | 2000-2011 |
| Guanaco | 7090643.3 | 475206 | González et al. (2013) | Cordillera | 2000-2011 |
| Guanaco | 6969143.3 | 475706 | González et al. (2013) | Cordillera | 2000-2011 |
| Guanaco | 6979643.3 | 475706 | González et al. (2013) | Cordillera | 2000-2011 |
| Guanaco | 6984143.3 | 475706 | González et al. (2013) | Cordillera | 2000-2011 |
| Guanaco | 6983143.3 | 476706 | González et al. (2013) | Cordillera | 2000-2011 |
| Guanaco | 6983643.3 | 476706 | González et al. (2013) | Cordillera | 2000-2011 |
| Guanaco | 7026643.3 | 477206 | González et al. (2013) | Cordillera | 2000-2011 |
| Guanaco | 7027143.3 | 479206 | González et al. (2013) | Cordillera | 2000-2011 |
| Guanaco | 6961143.3 | 479706 | González et al. (2013) | Cordillera | 2000-2011 |
| Guanaco | 7030143.3 | 480206 | Feces - Sighting | Cordillera | 2012 - 2014 |
| Guanaco | 6952643.3 | 481706 | Feces - Sighting | Cordillera | 2012 - 2014 |
| Guanaco | 7030143.3 | 481706 | Feces - Sighting | Cordillera | 2012 - 2014 |
| Guanaco | 7032143.3 | 485206 | Feces - Sighting | Cordillera | 2012 - 2014 |
| Guanaco | 7045143.3 | 486706 | Feces - Sighting | Cordillera | 2012 - 2014 |
| Guanaco | 6947643.3 | 492206 | González et al. (2013) | Cordillera | 2000-2011 |
| Guanaco | 6948643.3 | 492706 | González et al. (2013) | Cordillera | 2000-2011 |
| Guanaco | 7048643.3 | 495206 | Feces - Sighting | Cordillera | 2012 - 2014 |
| Guanaco | 7064643.3 | 496706 | Bonacic et al. (2014) | Cordillera | 2014 |
| Guanaco | 7067643.3 | 513706 | González et al. (2013) | Cordillera | 2000-2011 |
| Guanaco | 6832143.3 | 385706 | SEIA (2011) | El Morro | 2007 |
| Guanaco | 6833143.3 | 392206 | Researchers contribution | El Morro | 2008-2012 |
| Guanaco | 6843143.3 | 397206 | Bonacic et al. (2014) | El Morro | 2014 |
| Guanaco | 6834143.3 | 398206 | SEIA (2011) | El Morro | 2007 |
| Guanaco | 6834143.3 | 407206 | SEIA (2011) | El Morro | 2007 |
| Guanaco | 6834143.3 | 407706 | SEIA (2011) | El Morro | 2007 |
| Guanaco | 6833643.3 | 408706 | SEIA (2011) | El Morro | 2007 |
| Guanaco | 6831143.3 | 411206 | SEIA (2011) | El Morro | 2007 |
| Guanaco | 6833143.3 | 411206 | SEIA (2011) | El Morro | 2007 |
| Guanaco | 6699143.3 | 410206 | SEIA (1994) | El Tambo stream | 1994 |
| Guanaco | 6697787 | 409035 | Researchers contribution | El Tambo stream | 2002-2008 |
| Guanaco | 6634067.6 | 383187.2 | Researchers contribution | Estero Derecho nature sanctuary | 2002-2008 |
| Guanaco | 6877843.3 | 291606 | Feces - Sighting | Llanos de Challe National Park | 2012 - 2014 |
| Guanaco | 6877843.3 | 292306 | Feces - Sighting | Llanos de Challe National Park | 2012 - 2014 |
| Guanaco | 6877843.3 | 293006 | Feces - Sighting | Llanos de Challe National Park | 2012 - 2014 |
| Guanaco | 6879943.3 | 293006 | Feces - Sighting | Llanos de Challe National Park | 2012 - 2014 |
| Guanaco | 6879943.3 | 293706 | Feces - Sighting | Llanos de Challe National Park | 2012 - 2014 |
| Guanaco | 6896743.3 | 293706 | Feces - Sighting | Llanos de Challe National Park | 2012 - 2014 |
| Guanaco | 6879943.3 | 294406 | Feces - Sighting | Llanos de Challe National Park | 2012 - 2014 |
| Guanaco | 6895343.3 | 294406 | Feces - Sighting | Llanos de Challe National Park | 2012 - 2014 |
| Guanaco | 6896043.3 | 294406 | Feces - Sighting | Llanos de Challe National Park | 2012 - 2014 |
| Guanaco | 6896743.3 | 294406 | Feces - Sighting | Llanos de Challe National Park | 2012 - 2014 |
| Guanaco | 6888343.3 | 296506 | Feces - Sighting | Llanos de Challe National Park | 2012 - 2014 |
| Guanaco | 6881343.3 | 297206 | Feces - Sighting | Llanos de Challe National Park | 2012 - 2014 |
| Guanaco | 6886943.3 | 297206 | Feces - Sighting | Llanos de Challe National Park | 2012 - 2014 |
| Guanaco | 6887643.3 | 297206 | Feces - Sighting | Llanos de Challe National Park | 2012 - 2014 |
| Guanaco | 6888343.3 | 297206 | Feces - Sighting | Llanos de Challe National Park | 2012 - 2014 |
| Guanaco | 6874343.3 | 297906 | Feces - Sighting | Llanos de Challe National Park | 2012 - 2014 |
| Guanaco | 6881343.3 | 297906 | Feces - Sighting | Llanos de Challe National Park | 2012 - 2014 |
| Guanaco | 6887643.3 | 297906 | Feces - Sighting | Llanos de Challe National Park | 2012 - 2014 |
| Guanaco | 6878543.3 | 298606 | Feces - Sighting | Llanos de Challe National Park | 2012 - 2014 |
| Guanaco | 6877843.3 | 299306 | Feces - Sighting | Llanos de Challe National Park | 2012 - 2014 |
| Guanaco | 6878543.3 | 299306 | Feces - Sighting | Llanos de Challe National Park | 2012 - 2014 |
| Guanaco | 6876443.3 | 300006 | Feces - Sighting | Llanos de Challe National Park | 2012 - 2014 |
| Guanaco | 6877143.3 | 300006 | Feces - Sighting | Llanos de Challe National Park | 2012 - 2014 |
| Guanaco | 6877843.3 | 300006 | Feces - Sighting | Llanos de Challe National Park | 2012 - 2014 |
| Guanaco | 6875743.3 | 300706 | Feces - Sighting | Llanos de Challe National Park | 2012 - 2014 |
| Guanaco | 6875043.3 | 301406 | Feces - Sighting | Llanos de Challe National Park | 2012 - 2014 |
| Guanaco | 6875743.3 | 301406 | Feces - Sighting | Llanos de Challe National Park | 2012 - 2014 |
| Guanaco | 6876443.3 | 301406 | Feces - Sighting | Llanos de Challe National Park | 2012 - 2014 |
| Guanaco | 6877143.3 | 301406 | Feces - Sighting | Llanos de Challe National Park | 2012 - 2014 |
| Guanaco | 6877843.3 | 301406 | Feces - Sighting | Llanos de Challe National Park | 2012 - 2014 |
| Guanaco | 6878543.3 | 301406 | Feces - Sighting | Llanos de Challe National Park | 2012 - 2014 |
| Guanaco | 6874343.3 | 302106 | Feces - Sighting | Llanos de Challe National Park | 2012 - 2014 |
| Guanaco | 6875043.3 | 302106 | Feces - Sighting | Llanos de Challe National Park | 2012 - 2014 |
| Guanaco | 6874343.3 | 302806 | Feces - Sighting | Llanos de Challe National Park | 2012 - 2014 |
| Guanaco | 6875043.3 | 302806 | Feces - Sighting | Llanos de Challe National Park | 2012 - 2014 |
| Guanaco | 6875743.3 | 302806 | Feces - Sighting | Llanos de Challe National Park | 2012 - 2014 |
| Guanaco | 6874343.3 | 303506 | Feces - Sighting | Llanos de Challe National Park | 2012 - 2014 |
| Guanaco | 6875743.3 | 303506 | Feces - Sighting | Llanos de Challe National Park | 2012 - 2014 |
| Guanaco | 6869643.3 | 288206 | Feces - Sighting | Llanos de Challe National Park | 2012 - 2014 |
| Guanaco | 6886643.3 | 288206 | Feces - Sighting | Llanos de Challe National Park | 2012 - 2014 |
| Guanaco | 6871143.3 | 288706 | Feces - Sighting | Llanos de Challe National Park | 2012 - 2014 |
| Guanaco | 6886643.3 | 288706 | Feces - Sighting | Llanos de Challe National Park | 2012 - 2014 |
| Guanaco | 6877643.3 | 289706 | Feces - Sighting | Llanos de Challe National Park | 2012 - 2014 |
| Guanaco | 6878143.3 | 289706 | Feces - Sighting | Llanos de Challe National Park | 2012 - 2014 |
| Guanaco | 6896143.3 | 292206 | Feces - Sighting | Llanos de Challe National Park | 2012 - 2014 |
| Guanaco | 6889143.3 | 293206 | Feces - Sighting | Llanos de Challe National Park | 2012 - 2014 |
| Guanaco | 6869143.3 | 294706 | Feces - Sighting | Llanos de Challe National Park | 2012 - 2014 |
| Guanaco | 6869643.3 | 294706 | Feces - Sighting | Llanos de Challe National Park | 2012 - 2014 |
| Guanaco | 6888143.3 | 295206 | Feces - Sighting | Llanos de Challe National Park | 2012 - 2014 |
| Guanaco | 6888643.3 | 295206 | Feces - Sighting | Llanos de Challe National Park | 2012 - 2014 |
| Guanaco | 6870143.3 | 295706 | Feces - Sighting | Llanos de Challe National Park | 2012 - 2014 |
| Guanaco | 6871643.3 | 296206 | Feces - Sighting | Llanos de Challe National Park | 2012 - 2014 |
| Guanaco | 6872143.3 | 296206 | Feces - Sighting | Llanos de Challe National Park | 2012 - 2014 |
| Guanaco | 6871643.3 | 296706 | Feces - Sighting | Llanos de Challe National Park | 2012 - 2014 |
| Guanaco | 6880643.3 | 298206 | Feces - Sighting | Llanos de Challe National Park | 2012 - 2014 |
| Guanaco | 6882643.3 | 298206 | Feces - Sighting | Llanos de Challe National Park | 2012 - 2014 |
| Guanaco | 6883143.3 | 298206 | Feces - Sighting | Llanos de Challe National Park | 2012 - 2014 |
| Guanaco | 6879143.3 | 298706 | Feces - Sighting | Llanos de Challe National Park | 2012 - 2014 |
| Guanaco | 6880143.3 | 299706 | Feces - Sighting | Llanos de Challe National Park | 2012 - 2014 |
| Guanaco | 6879643.3 | 300706 | Feces - Sighting | Llanos de Challe National Park | 2012 - 2014 |
| Guanaco | 6884143.3 | 300706 | Feces - Sighting | Llanos de Challe National Park | 2012 - 2014 |
| Guanaco | 6884143.3 | 301706 | Feces - Sighting | Llanos de Challe National Park | 2012 - 2014 |
| Guanaco | 6890143.3 | 301706 | Feces - Sighting | Llanos de Challe National Park | 2012 - 2014 |
| Guanaco | 6890643.3 | 301706 | Feces - Sighting | Llanos de Challe National Park | 2012 - 2014 |
| Guanaco | 6889143.3 | 302706 | Feces - Sighting | Llanos de Challe National Park | 2012 - 2014 |
| Guanaco | 6884643.3 | 307206 | Feces - Sighting | Llanos de Challe National Park | 2012 - 2014 |
| Guanaco | 6751143.3 | 276906 | Collar GPS_No.15 | Los Choros | 2012 - 2015 |
| Guanaco | 6751843.3 | 278306 | Collar GPS_No.15 | Los Choros | 2012 - 2015 |
| Guanaco | 6752543.3 | 278306 | Collar GPS_No.15 | Los Choros | 2012 - 2015 |
| Guanaco | 6755343.3 | 278306 | Collar GPS_No.15 | Los Choros | 2012 - 2015 |
| Guanaco | 6749043.3 | 279006 | Feces - Sighting | Los Choros | 2012 - 2014 |
| Guanaco | 6749743.3 | 279006 | Feces - Sighting | Los Choros | 2012 - 2014 |
| Guanaco | 6750443.3 | 279006 | Feces - Sighting | Los Choros | 2012 - 2014 |
| Guanaco | 6753943.3 | 279006 | Collar GPS_No.15 | Los Choros | 2012 - 2015 |
| Guanaco | 6754643.3 | 279006 | Collar GPS_No.15 | Los Choros | 2012 - 2015 |
| Guanaco | 6755343.3 | 279006 | Collar GPS_No.15 | Los Choros | 2012 - 2015 |
| Guanaco | 6756043.3 | 279006 | Collar GPS_No.15 | Los Choros | 2012 - 2015 |
| Guanaco | 6756743.3 | 279006 | Collar GPS_No.15 | Los Choros | 2012 - 2015 |
| Guanaco | 6757443.3 | 279006 | Collar GPS_No.15 | Los Choros | 2012 - 2015 |
| Guanaco | 6758143.3 | 279006 | Collar GPS_No.15 | Los Choros | 2012 - 2015 |
| Guanaco | 6758843.3 | 279006 | Collar GPS_No.15 | Los Choros | 2012 - 2015 |
| Guanaco | 6760243.3 | 279006 | Collar GPS_No.15 | Los Choros | 2012 - 2015 |
| Guanaco | 6750443.3 | 279706 | Feces - Sighting | Los Choros | 2012 - 2014 |
| Guanaco | 6751143.3 | 279706 | Feces - Sighting | Los Choros | 2012 - 2014 |
| Guanaco | 6751843.3 | 279706 | Feces - Sighting | Los Choros | 2012 - 2014 |
| Guanaco | 6753943.3 | 279706 | Collar GPS_No.15 | Los Choros | 2012 - 2015 |
| Guanaco | 6754643.3 | 279706 | Collar GPS_No.15 | Los Choros | 2012 - 2015 |
| Guanaco | 6755343.3 | 279706 | Collar GPS_No.15 | Los Choros | 2012 - 2015 |
| Guanaco | 6756043.3 | 279706 | Collar GPS_No.15 | Los Choros | 2012 - 2015 |
| Guanaco | 6757443.3 | 279706 | Collar GPS_No.15 | Los Choros | 2012 - 2015 |
| Guanaco | 6750443.3 | 280406 | Feces - Sighting | Los Choros | 2012 - 2014 |
| Guanaco | 6751143.3 | 280406 | Feces - Sighting | Los Choros | 2012 - 2014 |
| Guanaco | 6751843.3 | 280406 | Feces - Sighting | Los Choros | 2012 - 2014 |
| Guanaco | 6753943.3 | 280406 | Collar GPS_No.15 | Los Choros | 2012 - 2015 |
| Guanaco | 6754643.3 | 280406 | Collar GPS_No.15 | Los Choros | 2012 - 2015 |
| Guanaco | 6755343.3 | 280406 | Collar GPS_No.15 | Los Choros | 2012 - 2015 |
| Guanaco | 6757443.3 | 280406 | Collar GPS_No.15 | Los Choros | 2012 - 2015 |
| Guanaco | 6751143.3 | 281106 | Feces - Sighting | Los Choros | 2012 - 2014 |
| Guanaco | 6751843.3 | 281106 | Feces - Sighting | Los Choros | 2012 - 2014 |
| Guanaco | 6752543.3 | 281106 | Feces - Sighting | Los Choros | 2012 - 2014 |
| Guanaco | 6753243.3 | 281106 | Collar GPS_No.15 | Los Choros | 2012 - 2015 |
| Guanaco | 6753943.3 | 281106 | Feces - Sighting | Los Choros | 2012 - 2014 |
| Guanaco | 6754643.3 | 281106 | Feces - Sighting | Los Choros | 2012 - 2014 |
| Guanaco | 6755343.3 | 281106 | Feces - Sighting | Los Choros | 2012 - 2014 |
| Guanaco | 6750443.3 | 281806 | Collar GPS_No.15 | Los Choros | 2012 - 2015 |
| Guanaco | 6751143.3 | 281806 | Feces - Sighting | Los Choros | 2012 - 2014 |
| Guanaco | 6751843.3 | 281806 | Feces - Sighting | Los Choros | 2012 - 2014 |
| Guanaco | 6752543.3 | 281806 | Feces - Sighting | Los Choros | 2012 - 2014 |
| Guanaco | 6753243.3 | 281806 | Feces - Sighting | Los Choros | 2012 - 2014 |
| Guanaco | 6753943.3 | 281806 | Feces - Sighting | Los Choros | 2012 - 2014 |
| Guanaco | 6754643.3 | 281806 | Feces - Sighting | Los Choros | 2012 - 2014 |
| Guanaco | 6755343.3 | 281806 | Feces - Sighting | Los Choros | 2012 - 2014 |
| Guanaco | 6756043.3 | 281806 | Collar GPS_No.15 | Los Choros | 2012 - 2015 |
| Guanaco | 6760243.3 | 281806 | Collar GPS_No.15 | Los Choros | 2012 - 2015 |
| Guanaco | 6748343.3 | 282506 | Collar GPS_No.15 | Los Choros | 2012 - 2015 |
| Guanaco | 6749743.3 | 282506 | Collar GPS_No.15 | Los Choros | 2012 - 2015 |
| Guanaco | 6751143.3 | 282506 | Feces - Sighting | Los Choros | 2012 - 2014 |
| Guanaco | 6751843.3 | 282506 | Feces - Sighting | Los Choros | 2012 - 2014 |
| Guanaco | 6752543.3 | 282506 | Feces - Sighting | Los Choros | 2012 - 2014 |
| Guanaco | 6753243.3 | 282506 | Feces - Sighting | Los Choros | 2012 - 2014 |
| Guanaco | 6753943.3 | 282506 | Feces - Sighting | Los Choros | 2012 - 2014 |
| Guanaco | 6754643.3 | 282506 | Feces - Sighting | Los Choros | 2012 - 2014 |
| Guanaco | 6755343.3 | 282506 | Feces - Sighting | Los Choros | 2012 - 2014 |
| Guanaco | 6756043.3 | 282506 | Collar GPS_No.15 | Los Choros | 2012 - 2015 |
| Guanaco | 6756743.3 | 282506 | Collar GPS_No.15 | Los Choros | 2012 - 2015 |
| Guanaco | 6757443.3 | 282506 | Collar GPS_No.15 | Los Choros | 2012 - 2015 |
| Guanaco | 6758143.3 | 282506 | Collar GPS_No.15 | Los Choros | 2012 - 2015 |
| Guanaco | 6758843.3 | 282506 | Collar GPS_No.15 | Los Choros | 2012 - 2015 |
| Guanaco | 6748343.3 | 283206 | Collar GPS_No.15 | Los Choros | 2012 - 2015 |
| Guanaco | 6750443.3 | 283206 | Collar GPS_No.15 | Los Choros | 2012 - 2015 |
| Guanaco | 6751843.3 | 283206 | Feces - Sighting | Los Choros | 2012 - 2014 |
| Guanaco | 6752543.3 | 283206 | Feces - Sighting | Los Choros | 2012 - 2014 |
| Guanaco | 6753243.3 | 283206 | Feces - Sighting | Los Choros | 2012 - 2014 |
| Guanaco | 6753943.3 | 283206 | Feces - Sighting | Los Choros | 2012 - 2014 |
| Guanaco | 6755343.3 | 283206 | Feces - Sighting | Los Choros | 2012 - 2014 |
| Guanaco | 6756043.3 | 283206 | Collar GPS_No.15 | Los Choros | 2012 - 2015 |
| Guanaco | 6756743.3 | 283206 | Collar GPS_No.15 | Los Choros | 2012 - 2015 |
| Guanaco | 6757443.3 | 283206 | Collar GPS_No.15 | Los Choros | 2012 - 2015 |
| Guanaco | 6758143.3 | 283206 | Collar GPS_No.15 | Los Choros | 2012 - 2015 |
| Guanaco | 6748343.3 | 283906 | Collar GPS_No.15 | Los Choros | 2012 - 2015 |
| Guanaco | 6749043.3 | 283906 | Collar GPS_No.15 | Los Choros | 2012 - 2015 |
| Guanaco | 6751843.3 | 283906 | Feces - Sighting | Los Choros | 2012 - 2014 |
| Guanaco | 6752543.3 | 283906 | Feces - Sighting | Los Choros | 2012 - 2014 |
| Guanaco | 6753243.3 | 283906 | Feces - Sighting | Los Choros | 2012 - 2014 |
| Guanaco | 6753943.3 | 283906 | Feces - Sighting | Los Choros | 2012 - 2014 |
| Guanaco | 6754643.3 | 283906 | Feces - Sighting | Los Choros | 2012 - 2014 |
| Guanaco | 6755343.3 | 283906 | Feces - Sighting | Los Choros | 2012 - 2014 |
| Guanaco | 6756043.3 | 283906 | Collar GPS_No.15 | Los Choros | 2012 - 2015 |
| Guanaco | 6756743.3 | 283906 | Collar GPS_No.15 | Los Choros | 2012 - 2015 |
| Guanaco | 6757443.3 | 283906 | Collar GPS_No.15 | Los Choros | 2012 - 2015 |
| Guanaco | 6758143.3 | 283906 | Collar GPS_No.15 | Los Choros | 2012 - 2015 |
| Guanaco | 6759543.3 | 283906 | Collar GPS_No.15 | Los Choros | 2012 - 2015 |
| Guanaco | 6760243.3 | 283906 | Collar GPS_No.15 | Los Choros | 2012 - 2015 |
| Guanaco | 6751143.3 | 284606 | Feces - Sighting | Los Choros | 2012 - 2014 |
| Guanaco | 6751843.3 | 284606 | Feces - Sighting | Los Choros | 2012 - 2014 |
| Guanaco | 6752543.3 | 284606 | Feces - Sighting | Los Choros | 2012 - 2014 |
| Guanaco | 6753243.3 | 284606 | Feces - Sighting | Los Choros | 2012 - 2014 |
| Guanaco | 6753943.3 | 284606 | Feces - Sighting | Los Choros | 2012 - 2014 |
| Guanaco | 6754643.3 | 284606 | Feces - Sighting | Los Choros | 2012 - 2014 |
| Guanaco | 6755343.3 | 284606 | Feces - Sighting | Los Choros | 2012 - 2014 |
| Guanaco | 6756043.3 | 284606 | Collar GPS_No.15 | Los Choros | 2012 - 2015 |
| Guanaco | 6756743.3 | 284606 | Collar GPS_No.15 | Los Choros | 2012 - 2015 |
| Guanaco | 6758843.3 | 284606 | Collar GPS_No.15 | Los Choros | 2012 - 2015 |
| Guanaco | 6751843.3 | 285306 | Feces - Sighting | Los Choros | 2012 - 2014 |
| Guanaco | 6752543.3 | 285306 | Feces - Sighting | Los Choros | 2012 - 2014 |
| Guanaco | 6753243.3 | 285306 | Feces - Sighting | Los Choros | 2012 - 2014 |
| Guanaco | 6753943.3 | 285306 | Feces - Sighting | Los Choros | 2012 - 2014 |
| Guanaco | 6754643.3 | 285306 | Feces - Sighting | Los Choros | 2012 - 2014 |
| Guanaco | 6755343.3 | 285306 | Feces - Sighting | Los Choros | 2012 - 2014 |
| Guanaco | 6756043.3 | 285306 | Collar GPS_No.15 | Los Choros | 2012 - 2015 |
| Guanaco | 6756743.3 | 285306 | Collar GPS_No.15 | Los Choros | 2012 - 2015 |
| Guanaco | 6757443.3 | 285306 | Collar GPS_No.15 | Los Choros | 2012 - 2015 |
| Guanaco | 6749043.3 | 286006 | Collar GPS_No.15 | Los Choros | 2012 - 2015 |
| Guanaco | 6749743.3 | 286006 | Collar GPS_No.15 | Los Choros | 2012 - 2015 |
| Guanaco | 6750443.3 | 286006 | Collar GPS_No.15 | Los Choros | 2012 - 2015 |
| Guanaco | 6751143.3 | 286006 | Feces - Sighting | Los Choros | 2012 - 2014 |
| Guanaco | 6751843.3 | 286006 | Feces - Sighting | Los Choros | 2012 - 2014 |
| Guanaco | 6752543.3 | 286006 | Feces - Sighting | Los Choros | 2012 - 2014 |
| Guanaco | 6753243.3 | 286006 | Feces - Sighting | Los Choros | 2012 - 2014 |
| Guanaco | 6753943.3 | 286006 | Feces - Sighting | Los Choros | 2012 - 2014 |
| Guanaco | 6754643.3 | 286006 | Feces - Sighting | Los Choros | 2012 - 2014 |
| Guanaco | 6755343.3 | 286006 | Feces - Sighting | Los Choros | 2012 - 2014 |
| Guanaco | 6756043.3 | 286006 | Collar GPS_No.15 | Los Choros | 2012 - 2015 |
| Guanaco | 6756743.3 | 286006 | Collar GPS_No.15 | Los Choros | 2012 - 2015 |
| Guanaco | 6757443.3 | 286006 | Collar GPS_No.15 | Los Choros | 2012 - 2015 |
| Guanaco | 6749043.3 | 286706 | Collar GPS_No.15 | Los Choros | 2012 - 2015 |
| Guanaco | 6749743.3 | 286706 | Collar GPS_No.15 | Los Choros | 2012 - 2015 |
| Guanaco | 6750443.3 | 286706 | Collar GPS_No.15 | Los Choros | 2012 - 2015 |
| Guanaco | 6751843.3 | 286706 | Feces - Sighting | Los Choros | 2012 - 2014 |
| Guanaco | 6752543.3 | 286706 | Feces - Sighting | Los Choros | 2012 - 2014 |
| Guanaco | 6753243.3 | 286706 | Feces - Sighting | Los Choros | 2012 - 2014 |
| Guanaco | 6753943.3 | 286706 | Feces - Sighting | Los Choros | 2012 - 2014 |
| Guanaco | 6754643.3 | 286706 | Feces - Sighting | Los Choros | 2012 - 2014 |
| Guanaco | 6755343.3 | 286706 | Feces - Sighting | Los Choros | 2012 - 2014 |
| Guanaco | 6756743.3 | 286706 | Collar GPS_No.15 | Los Choros | 2012 - 2015 |
| Guanaco | 6749043.3 | 287406 | Collar GPS_No.15 | Los Choros | 2012 - 2015 |
| Guanaco | 6749743.3 | 287406 | Collar GPS_No.15 | Los Choros | 2012 - 2015 |
| Guanaco | 6750443.3 | 287406 | Collar GPS_No.15 | Los Choros | 2012 - 2015 |
| Guanaco | 6751143.3 | 287406 | Collar GPS_No.15 | Los Choros | 2012 - 2015 |
| Guanaco | 6751843.3 | 287406 | Collar GPS_No.15 | Los Choros | 2012 - 2015 |
| Guanaco | 6752543.3 | 287406 | Collar GPS_No.15 | Los Choros | 2012 - 2015 |
| Guanaco | 6753243.3 | 287406 | Feces - Sighting | Los Choros | 2012 - 2014 |
| Guanaco | 6753943.3 | 287406 | Feces - Sighting | Los Choros | 2012 - 2014 |
| Guanaco | 6757443.3 | 287406 | Collar GPS_No.15 | Los Choros | 2012 - 2015 |
| Guanaco | 6749743.3 | 288106 | Collar GPS_No.15 | Los Choros | 2012 - 2015 |
| Guanaco | 6750443.3 | 288106 | Collar GPS_No.15 | Los Choros | 2012 - 2015 |
| Guanaco | 6751143.3 | 288106 | Collar GPS_No.15 | Los Choros | 2012 - 2015 |
| Guanaco | 6751843.3 | 288106 | Collar GPS_No.15 | Los Choros | 2012 - 2015 |
| Guanaco | 6752543.3 | 288106 | Collar GPS_No.15 | Los Choros | 2012 - 2015 |
| Guanaco | 6753243.3 | 288106 | Collar GPS_No.15 | Los Choros | 2012 - 2015 |
| Guanaco | 6755343.3 | 288106 | Feces - Sighting | Los Choros | 2012 - 2014 |
| Guanaco | 6756043.3 | 288106 | Collar GPS_No.15 | Los Choros | 2012 - 2015 |
| Guanaco | 6749743.3 | 288806 | Collar GPS_No.15 | Los Choros | 2012 - 2015 |
| Guanaco | 6750443.3 | 288806 | Collar GPS_No.15 | Los Choros | 2012 - 2015 |
| Guanaco | 6751143.3 | 288806 | Collar GPS_No.15 | Los Choros | 2012 - 2015 |
| Guanaco | 6751843.3 | 288806 | Collar GPS_No.15 | Los Choros | 2012 - 2015 |
| Guanaco | 6752543.3 | 288806 | Collar GPS_No.15 | Los Choros | 2012 - 2015 |
| Guanaco | 6753243.3 | 288806 | Collar GPS_No.15 | Los Choros | 2012 - 2015 |
| Guanaco | 6753943.3 | 288806 | Collar GPS_No.15 | Los Choros | 2012 - 2015 |
| Guanaco | 6750443.3 | 289506 | Collar GPS_No.15 | Los Choros | 2012 - 2015 |
| Guanaco | 6751143.3 | 289506 | Collar GPS_No.15 | Los Choros | 2012 - 2015 |
| Guanaco | 6751843.3 | 289506 | Collar GPS_No.15 | Los Choros | 2012 - 2015 |
| Guanaco | 6752543.3 | 289506 | Collar GPS_No.15 | Los Choros | 2012 - 2015 |
| Guanaco | 6753943.3 | 289506 | Collar GPS_No.15 | Los Choros | 2012 - 2015 |
| Guanaco | 6754643.3 | 289506 | Feces - Sighting | Los Choros | 2012 - 2014 |
| Guanaco | 6755343.3 | 289506 | Feces - Sighting | Los Choros | 2012 - 2014 |
| Guanaco | 6750443.3 | 290206 | Collar GPS_No.15 | Los Choros | 2012 - 2015 |
| Guanaco | 6751143.3 | 290206 | Collar GPS_No.15 | Los Choros | 2012 - 2015 |
| Guanaco | 6751843.3 | 290206 | Collar GPS_No.15 | Los Choros | 2012 - 2015 |
| Guanaco | 6751843.3 | 290906 | Collar GPS_No.15 | Los Choros | 2012 - 2015 |
| Guanaco | 6752543.3 | 290906 | Feces - Sighting | Los Choros | 2012 - 2014 |
| Guanaco | 7005143.3 | 476706 | Feces - Sighting | Nevado Tres Cruces National Park | 2012 - 2014 |
| Guanaco | 6978643.3 | 478706 | González et al. (2013) | Nevado Tres Cruces National Park | 2000-2011 |
| Guanaco | 6981643.3 | 479206 | González et al. (2013) | Nevado Tres Cruces National Park | 2000-2011 |
| Guanaco | 6981143.3 | 480206 | González et al. (2013) | Nevado Tres Cruces National Park | 2000-2011 |
| Guanaco | 7004143.3 | 482706 | Bonacic et al. (2014) | Nevado Tres Cruces National Park | 2014 |
| Guanaco | 6960143.3 | 491706 | Feces - Sighting | Nevado Tres Cruces National Park | 2012 - 2014 |
| Guanaco | 6954143.3 | 494706 | González et al. (2013) | Nevado Tres Cruces National Park | 2000-2011 |
| Guanaco | 6962143.3 | 495206 | González et al. (2013) | Nevado Tres Cruces National Park | 2000-2011 |
| Guanaco | 6973143.3 | 495206 | Feces - Sighting | Nevado Tres Cruces National Park | 2012 - 2014 |
| Guanaco | 6990643.3 | 495206 | Feces - Sighting | Nevado Tres Cruces National Park | 2012 - 2014 |
| Guanaco | 6960143.3 | 496206 | González et al. (2013) | Nevado Tres Cruces National Park | 2000-2011 |
| Guanaco | 6990643.3 | 497206 | Feces - Sighting | Nevado Tres Cruces National Park | 2012 - 2014 |
| Guanaco | 6980143.3 | 498206 | Feces - Sighting | Nevado Tres Cruces National Park | 2012 - 2014 |
| Guanaco | 6991143.3 | 498706 | Feces - Sighting | Nevado Tres Cruces National Park | 2012 - 2014 |
| Guanaco | 6971143.3 | 499706 | González et al. (2013) | Nevado Tres Cruces National Park | 2000-2011 |
| Guanaco | 6996643.3 | 499706 | González et al. (2013) | Nevado Tres Cruces National Park | 2000-2011 |
| Guanaco | 7004643.3 | 506706 | González et al. (2013) | Nevado Tres Cruces National Park | 2000-2011 |
| Guanaco | 6911443.3 | 352506 | Feces - Sighting | Oso Negro | 2014 |
| Guanaco | 6921943.3 | 352506 | Feces - Sighting | Oso Negro | 2014 |
| Guanaco | 6922643.3 | 352506 | Feces - Sighting | Oso Negro | 2014 |
| Guanaco | 6923343.3 | 352506 | Feces - Sighting | Oso Negro | 2014 |
| Guanaco | 6914243.3 | 353206 | Feces - Sighting | Oso Negro | 2014 |
| Guanaco | 6921943.3 | 353206 | Feces - Sighting | Oso Negro | 2014 |
| Guanaco | 6922643.3 | 353206 | Feces - Sighting | Oso Negro | 2014 |
| Guanaco | 6923343.3 | 353206 | Feces - Sighting | Oso Negro | 2014 |
| Guanaco | 6924043.3 | 353206 | Feces - Sighting | Oso Negro | 2014 |
| Guanaco | 6911443.3 | 353906 | Feces - Sighting | Oso Negro | 2014 |
| Guanaco | 6912143.3 | 353906 | Feces - Sighting | Oso Negro | 2014 |
| Guanaco | 6912843.3 | 353906 | Feces - Sighting | Oso Negro | 2014 |
| Guanaco | 6913543.3 | 353906 | Feces - Sighting | Oso Negro | 2014 |
| Guanaco | 6914243.3 | 353906 | Feces - Sighting | Oso Negro | 2014 |
| Guanaco | 6914943.3 | 353906 | Feces - Sighting | Oso Negro | 2014 |
| Guanaco | 6921243.3 | 353906 | Feces - Sighting | Oso Negro | 2014 |
| Guanaco | 6921943.3 | 353906 | Feces - Sighting | Oso Negro | 2014 |
| Guanaco | 6922643.3 | 353906 | Feces - Sighting | Oso Negro | 2014 |
| Guanaco | 6923343.3 | 353906 | Feces - Sighting | Oso Negro | 2014 |
| Guanaco | 6924043.3 | 353906 | Feces - Sighting | Oso Negro | 2014 |
| Guanaco | 6924743.3 | 353906 | Feces - Sighting | Oso Negro | 2014 |
| Guanaco | 6911443.3 | 354606 | Feces - Sighting | Oso Negro | 2014 |
| Guanaco | 6912843.3 | 354606 | Feces - Sighting | Oso Negro | 2014 |
| Guanaco | 6913543.3 | 354606 | Feces - Sighting | Oso Negro | 2014 |
| Guanaco | 6914243.3 | 354606 | Feces - Sighting | Oso Negro | 2014 |
| Guanaco | 6914943.3 | 354606 | Feces - Sighting | Oso Negro | 2014 |
| Guanaco | 6920543.3 | 354606 | Feces - Sighting | Oso Negro | 2014 |
| Guanaco | 6921943.3 | 354606 | Feces - Sighting | Oso Negro | 2014 |
| Guanaco | 6922643.3 | 354606 | Feces - Sighting | Oso Negro | 2014 |
| Guanaco | 6912143.3 | 355306 | Feces - Sighting | Oso Negro | 2014 |
| Guanaco | 6912843.3 | 355306 | Feces - Sighting | Oso Negro | 2014 |
| Guanaco | 6913543.3 | 355306 | Feces - Sighting | Oso Negro | 2014 |
| Guanaco | 6914943.3 | 355306 | Feces - Sighting | Oso Negro | 2014 |
| Guanaco | 6921943.3 | 355306 | Feces - Sighting | Oso Negro | 2014 |
| Guanaco | 6912143.3 | 356006 | Feces - Sighting | Oso Negro | 2014 |
| Guanaco | 6912843.3 | 356006 | Feces - Sighting | Oso Negro | 2014 |
| Guanaco | 6914243.3 | 356006 | Feces - Sighting | Oso Negro | 2014 |
| Guanaco | 6914943.3 | 356006 | Feces - Sighting | Oso Negro | 2014 |
| Guanaco | 6917043.3 | 356006 | Feces - Sighting | Oso Negro | 2014 |
| Guanaco | 6917743.3 | 356006 | Feces - Sighting | Oso Negro | 2014 |
| Guanaco | 6919143.3 | 356006 | Feces - Sighting | Oso Negro | 2014 |
| Guanaco | 6919843.3 | 356006 | Feces - Sighting | Oso Negro | 2014 |
| Guanaco | 6920543.3 | 356006 | Feces - Sighting | Oso Negro | 2014 |
| Guanaco | 6912843.3 | 356706 | Feces - Sighting | Oso Negro | 2014 |
| Guanaco | 6913543.3 | 356706 | Feces - Sighting | Oso Negro | 2014 |
| Guanaco | 6914943.3 | 356706 | Feces - Sighting | Oso Negro | 2014 |
| Guanaco | 6918443.3 | 356706 | Feces - Sighting | Oso Negro | 2014 |
| Guanaco | 6919143.3 | 356706 | Feces - Sighting | Oso Negro | 2014 |
| Guanaco | 6911443.3 | 357406 | Feces - Sighting | Oso Negro | 2014 |
| Guanaco | 6912143.3 | 357406 | Feces - Sighting | Oso Negro | 2014 |
| Guanaco | 6912843.3 | 357406 | Feces - Sighting | Oso Negro | 2014 |
| Guanaco | 6914943.3 | 357406 | Feces - Sighting | Oso Negro | 2014 |
| Guanaco | 6916343.3 | 357406 | Feces - Sighting | Oso Negro | 2014 |
| Guanaco | 6918443.3 | 357406 | Feces - Sighting | Oso Negro | 2014 |
| Guanaco | 6911443.3 | 358106 | Feces - Sighting | Oso Negro | 2014 |
| Guanaco | 6912143.3 | 358106 | Feces - Sighting | Oso Negro | 2014 |
| Guanaco | 6912843.3 | 358106 | Feces - Sighting | Oso Negro | 2014 |
| Guanaco | 6914243.3 | 358106 | Feces - Sighting | Oso Negro | 2014 |
| Guanaco | 6914943.3 | 358106 | Feces - Sighting | Oso Negro | 2014 |
| Guanaco | 6915643.3 | 358106 | Feces - Sighting | Oso Negro | 2014 |
| Guanaco | 6918443.3 | 358106 | Feces - Sighting | Oso Negro | 2014 |
| Guanaco | 6912843.3 | 358806 | Feces - Sighting | Oso Negro | 2014 |
| Guanaco | 6913543.3 | 358806 | Feces - Sighting | Oso Negro | 2014 |
| Guanaco | 6914243.3 | 358806 | Feces - Sighting | Oso Negro | 2014 |
| Guanaco | 6914943.3 | 358806 | Feces - Sighting | Oso Negro | 2014 |
| Guanaco | 6915643.3 | 358806 | Feces - Sighting | Oso Negro | 2014 |
| Guanaco | 6912843.3 | 359506 | Feces - Sighting | Oso Negro | 2014 |
| Guanaco | 6914243.3 | 359506 | Feces - Sighting | Oso Negro | 2014 |
| Guanaco | 6914943.3 | 359506 | Feces - Sighting | Oso Negro | 2014 |
| Guanaco | 6915643.3 | 359506 | Feces - Sighting | Oso Negro | 2014 |
| Guanaco | 6918443.3 | 359506 | Feces - Sighting | Oso Negro | 2014 |
| Guanaco | 6912843.3 | 360206 | Feces - Sighting | Oso Negro | 2014 |
| Guanaco | 6914243.3 | 360206 | Feces - Sighting | Oso Negro | 2014 |
| Guanaco | 6914943.3 | 360206 | Feces - Sighting | Oso Negro | 2014 |
| Guanaco | 6918443.3 | 360206 | Feces - Sighting | Oso Negro | 2014 |
| Guanaco | 6914243.3 | 360906 | Feces - Sighting | Oso Negro | 2014 |
| Guanaco | 6915643.3 | 360906 | Feces - Sighting | Oso Negro | 2014 |
| Guanaco | 6918443.3 | 360906 | Feces - Sighting | Oso Negro | 2014 |
| Guanaco | 6914943.3 | 361606 | Feces - Sighting | Oso Negro | 2014 |
| Guanaco | 6917743.3 | 361606 | Feces - Sighting | Oso Negro | 2014 |
| Guanaco | 7101643.3 | 335706 | Bonacic et al. (2014) | Pan de Azúcar National Park | 2014 |
| Guanaco | 7111143.3 | 335706 | González et al. (2013) | Pan de Azúcar National Park | 2000-2011 |
| Guanaco | 7101143.3 | 336206 | Bonacic et al. (2014) | Pan de Azúcar National Park | 2014 |
| Guanaco | 7101643.3 | 336206 | Bonacic et al. (2014) | Pan de Azúcar National Park | 2014 |
| Guanaco | 7110643.3 | 336206 | González et al. (2013) | Pan de Azúcar National Park | 2000-2011 |
| Guanaco | 7101143.3 | 337206 | Bonacic et al. (2014) | Pan de Azúcar National Park | 2014 |
| Guanaco | 7100143.3 | 338206 | Bonacic et al. (2014) | Pan de Azúcar National Park | 2014 |
| Guanaco | 7099143.3 | 339706 | Bonacic et al. (2014) | Pan de Azúcar National Park | 2014 |
| Guanaco | 7099143.3 | 341206 | Bonacic et al. (2014) | Pan de Azúcar National Park | 2014 |
| Guanaco | 7110643.3 | 342706 | González et al. (2013) | Pan de Azúcar National Park | 2000-2011 |
| Guanaco | 7117143.3 | 345706 | González et al. (2013) | Pan de Azúcar National Park | 2000-2011 |
| Guanaco | 7115643.3 | 346706 | González et al. (2013) | Pan de Azúcar National Park | 2000-2011 |
| Guanaco | 6484143.3 | 335706 | González et al. (2013) | Pelambres | 2000-2011 |
| Guanaco | 6486643.3 | 337206 | González et al. (2013) | Pelambres | 2000-2011 |
| Guanaco | 6485143.3 | 337706 | González et al. (2013) | Pelambres | 2000-2011 |
| Guanaco | 6486143.3 | 337706 | González et al. (2013) | Pelambres | 2000-2011 |
| Guanaco | 6483143.3 | 338206 | González et al. (2013) | Pelambres | 2000-2011 |
| Guanaco | 6483643.3 | 338206 | González et al. (2013) | Pelambres | 2000-2011 |
| Guanaco | 6483643.3 | 338706 | González et al. (2013) | Pelambres | 2000-2011 |
| Guanaco | 6486143.3 | 338706 | González et al. (2013) | Pelambres | 2000-2011 |
| Guanaco | 6483643.3 | 339206 | González et al. (2013) | Pelambres | 2000-2011 |
| Guanaco | 6483643.3 | 340206 | González et al. (2013) | Pelambres | 2000-2011 |
| Guanaco | 6484643.3 | 340206 | González et al. (2013) | Pelambres | 2000-2011 |
| Guanaco | 6485643.3 | 340206 | González et al. (2013) | Pelambres | 2000-2011 |
| Guanaco | 6491143.3 | 340206 | González et al. (2013) | Pelambres | 2000-2011 |
| Guanaco | 6484643.3 | 340706 | González et al. (2013) | Pelambres | 2000-2011 |
| Guanaco | 6487143.3 | 340706 | González et al. (2013) | Pelambres | 2000-2011 |
| Guanaco | 6487643.3 | 340706 | González et al. (2013) | Pelambres | 2000-2011 |
| Guanaco | 6482643.3 | 341206 | González et al. (2013) | Pelambres | 2000-2011 |
| Guanaco | 6488643.3 | 341206 | González et al. (2013) | Pelambres | 2000-2011 |
| Guanaco | 6493143.3 | 341206 | González et al. (2013) | Pelambres | 2000-2011 |
| Guanaco | 6476643.3 | 341706 | González et al. (2013) | Pelambres | 2000-2011 |
| Guanaco | 6485643.3 | 341706 | González et al. (2013) | Pelambres | 2000-2011 |
| Guanaco | 6487143.3 | 341706 | González et al. (2013) | Pelambres | 2000-2011 |
| Guanaco | 6491643.3 | 341706 | González et al. (2013) | Pelambres | 2000-2011 |
| Guanaco | 6484643.3 | 342206 | González et al. (2013) | Pelambres | 2000-2011 |
| Guanaco | 6486643.3 | 342206 | González et al. (2013) | Pelambres | 2000-2011 |
| Guanaco | 6487643.3 | 342206 | González et al. (2013) | Pelambres | 2000-2011 |
| Guanaco | 6491143.3 | 342206 | González et al. (2013) | Pelambres | 2000-2011 |
| Guanaco | 6482643.3 | 342706 | González et al. (2013) | Pelambres | 2000-2011 |
| Guanaco | 6487643.3 | 342706 | González et al. (2013) | Pelambres | 2000-2011 |
| Guanaco | 6491143.3 | 342706 | González et al. (2013) | Pelambres | 2000-2011 |
| Guanaco | 6484143.3 | 343206 | González et al. (2013) | Pelambres | 2000-2011 |
| Guanaco | 6485143.3 | 343206 | González et al. (2013) | Pelambres | 2000-2011 |
| Guanaco | 6476143.3 | 343706 | González et al. (2013) | Pelambres | 2000-2011 |
| Guanaco | 6483643.3 | 343706 | González et al. (2013) | Pelambres | 2000-2011 |
| Guanaco | 6484643.3 | 343706 | González et al. (2013) | Pelambres | 2000-2011 |
| Guanaco | 6485643.3 | 343706 | González et al. (2013) | Pelambres | 2000-2011 |
| Guanaco | 6486143.3 | 343706 | González et al. (2013) | Pelambres | 2000-2011 |
| Guanaco | 6486643.3 | 343706 | González et al. (2013) | Pelambres | 2000-2011 |
| Guanaco | 6485643.3 | 344206 | González et al. (2013) | Pelambres | 2000-2011 |
| Guanaco | 6488643.3 | 344206 | González et al. (2013) | Pelambres | 2000-2011 |
| Guanaco | 6484643.3 | 344706 | González et al. (2013) | Pelambres | 2000-2011 |
| Guanaco | 6485643.3 | 344706 | González et al. (2013) | Pelambres | 2000-2011 |
| Guanaco | 6486643.3 | 344706 | González et al. (2013) | Pelambres | 2000-2011 |
| Guanaco | 6487643.3 | 344706 | González et al. (2013) | Pelambres | 2000-2011 |
| Guanaco | 6488143.3 | 344706 | González et al. (2013) | Pelambres | 2000-2011 |
| Guanaco | 6488643.3 | 344706 | González et al. (2013) | Pelambres | 2000-2011 |
| Guanaco | 6489143.3 | 344706 | González et al. (2013) | Pelambres | 2000-2011 |
| Guanaco | 6495643.3 | 344706 | González et al. (2013) | Pelambres | 2000-2011 |
| Guanaco | 6479143.3 | 345206 | González et al. (2013) | Pelambres | 2000-2011 |
| Guanaco | 6479643.3 | 345206 | González et al. (2013) | Pelambres | 2000-2011 |
| Guanaco | 6483143.3 | 345206 | González et al. (2013) | Pelambres | 2000-2011 |
| Guanaco | 6484643.3 | 345206 | González et al. (2013) | Pelambres | 2000-2011 |
| Guanaco | 6485143.3 | 345206 | González et al. (2013) | Pelambres | 2000-2011 |
| Guanaco | 6487143.3 | 345206 | González et al. (2013) | Pelambres | 2000-2011 |
| Guanaco | 6487643.3 | 345206 | González et al. (2013) | Pelambres | 2000-2011 |
| Guanaco | 6488143.3 | 345206 | González et al. (2013) | Pelambres | 2000-2011 |
| Guanaco | 6489143.3 | 345206 | González et al. (2013) | Pelambres | 2000-2011 |
| Guanaco | 6494143.3 | 345206 | González et al. (2013) | Pelambres | 2000-2011 |
| Guanaco | 6481643.3 | 345706 | González et al. (2013) | Pelambres | 2000-2011 |
| Guanaco | 6484643.3 | 345706 | González et al. (2013) | Pelambres | 2000-2011 |
| Guanaco | 6485143.3 | 345706 | González et al. (2013) | Pelambres | 2000-2011 |
| Guanaco | 6485643.3 | 345706 | González et al. (2013) | Pelambres | 2000-2011 |
| Guanaco | 6486643.3 | 345706 | González et al. (2013) | Pelambres | 2000-2011 |
| Guanaco | 6488143.3 | 345706 | González et al. (2013) | Pelambres | 2000-2011 |
| Guanaco | 6488643.3 | 345706 | González et al. (2013) | Pelambres | 2000-2011 |
| Guanaco | 6489643.3 | 345706 | González et al. (2013) | Pelambres | 2000-2011 |
| Guanaco | 6490143.3 | 345706 | González et al. (2013) | Pelambres | 2000-2011 |
| Guanaco | 6491143.3 | 345706 | González et al. (2013) | Pelambres | 2000-2011 |
| Guanaco | 6478143.3 | 346206 | González et al. (2013) | Pelambres | 2000-2011 |
| Guanaco | 6480643.3 | 346206 | González et al. (2013) | Pelambres | 2000-2011 |
| Guanaco | 6481143.3 | 346206 | González et al. (2013) | Pelambres | 2000-2011 |
| Guanaco | 6481643.3 | 346206 | González et al. (2013) | Pelambres | 2000-2011 |
| Guanaco | 6482643.3 | 346206 | González et al. (2013) | Pelambres | 2000-2011 |
| Guanaco | 6483643.3 | 346206 | González et al. (2013) | Pelambres | 2000-2011 |
| Guanaco | 6484643.3 | 346206 | González et al. (2013) | Pelambres | 2000-2011 |
| Guanaco | 6487643.3 | 346206 | González et al. (2013) | Pelambres | 2000-2011 |
| Guanaco | 6488143.3 | 346206 | González et al. (2013) | Pelambres | 2000-2011 |
| Guanaco | 6488643.3 | 346206 | González et al. (2013) | Pelambres | 2000-2011 |
| Guanaco | 6489143.3 | 346206 | González et al. (2013) | Pelambres | 2000-2011 |
| Guanaco | 6489643.3 | 346206 | González et al. (2013) | Pelambres | 2000-2011 |
| Guanaco | 6490643.3 | 346206 | González et al. (2013) | Pelambres | 2000-2011 |
| Guanaco | 6481643.3 | 346706 | González et al. (2013) | Pelambres | 2000-2011 |
| Guanaco | 6482143.3 | 346706 | González et al. (2013) | Pelambres | 2000-2011 |
| Guanaco | 6482643.3 | 346706 | González et al. (2013) | Pelambres | 2000-2011 |
| Guanaco | 6484143.3 | 346706 | González et al. (2013) | Pelambres | 2000-2011 |
| Guanaco | 6487143.3 | 346706 | González et al. (2013) | Pelambres | 2000-2011 |
| Guanaco | 6487643.3 | 346706 | González et al. (2013) | Pelambres | 2000-2011 |
| Guanaco | 6488143.3 | 346706 | González et al. (2013) | Pelambres | 2000-2011 |
| Guanaco | 6488643.3 | 346706 | González et al. (2013) | Pelambres | 2000-2011 |
| Guanaco | 6489143.3 | 346706 | González et al. (2013) | Pelambres | 2000-2011 |
| Guanaco | 6489643.3 | 346706 | González et al. (2013) | Pelambres | 2000-2011 |
| Guanaco | 6490143.3 | 346706 | González et al. (2013) | Pelambres | 2000-2011 |
| Guanaco | 6491643.3 | 346706 | González et al. (2013) | Pelambres | 2000-2011 |
| Guanaco | 6496643.3 | 346706 | González et al. (2013) | Pelambres | 2000-2011 |
| Guanaco | 6480643.3 | 347206 | González et al. (2013) | Pelambres | 2000-2011 |
| Guanaco | 6483643.3 | 347206 | González et al. (2013) | Pelambres | 2000-2011 |
| Guanaco | 6486643.3 | 347206 | González et al. (2013) | Pelambres | 2000-2011 |
| Guanaco | 6487143.3 | 347206 | González et al. (2013) | Pelambres | 2000-2011 |
| Guanaco | 6488143.3 | 347206 | González et al. (2013) | Pelambres | 2000-2011 |
| Guanaco | 6488643.3 | 347206 | González et al. (2013) | Pelambres | 2000-2011 |
| Guanaco | 6491643.3 | 347206 | González et al. (2013) | Pelambres | 2000-2011 |
| Guanaco | 6492143.3 | 347206 | González et al. (2013) | Pelambres | 2000-2011 |
| Guanaco | 6492643.3 | 347206 | González et al. (2013) | Pelambres | 2000-2011 |
| Guanaco | 6493143.3 | 347206 | González et al. (2013) | Pelambres | 2000-2011 |
| Guanaco | 6478143.3 | 347706 | González et al. (2013) | Pelambres | 2000-2011 |
| Guanaco | 6480643.3 | 347706 | González et al. (2013) | Pelambres | 2000-2011 |
| Guanaco | 6481643.3 | 347706 | González et al. (2013) | Pelambres | 2000-2011 |
| Guanaco | 6483143.3 | 347706 | González et al. (2013) | Pelambres | 2000-2011 |
| Guanaco | 6485143.3 | 347706 | González et al. (2013) | Pelambres | 2000-2011 |
| Guanaco | 6487143.3 | 347706 | González et al. (2013) | Pelambres | 2000-2011 |
| Guanaco | 6488643.3 | 347706 | González et al. (2013) | Pelambres | 2000-2011 |
| Guanaco | 6491143.3 | 347706 | González et al. (2013) | Pelambres | 2000-2011 |
| Guanaco | 6491643.3 | 347706 | González et al. (2013) | Pelambres | 2000-2011 |
| Guanaco | 6492643.3 | 347706 | González et al. (2013) | Pelambres | 2000-2011 |
| Guanaco | 6493143.3 | 347706 | González et al. (2013) | Pelambres | 2000-2011 |
| Guanaco | 6480643.3 | 348206 | González et al. (2013) | Pelambres | 2000-2011 |
| Guanaco | 6483143.3 | 348206 | González et al. (2013) | Pelambres | 2000-2011 |
| Guanaco | 6484143.3 | 348206 | González et al. (2013) | Pelambres | 2000-2011 |
| Guanaco | 6486143.3 | 348206 | González et al. (2013) | Pelambres | 2000-2011 |
| Guanaco | 6491143.3 | 348206 | González et al. (2013) | Pelambres | 2000-2011 |
| Guanaco | 6491643.3 | 348206 | González et al. (2013) | Pelambres | 2000-2011 |
| Guanaco | 6492143.3 | 348206 | González et al. (2013) | Pelambres | 2000-2011 |
| Guanaco | 6492643.3 | 348206 | González et al. (2013) | Pelambres | 2000-2011 |
| Guanaco | 6493143.3 | 348206 | González et al. (2013) | Pelambres | 2000-2011 |
| Guanaco | 6481143.3 | 348706 | González et al. (2013) | Pelambres | 2000-2011 |
| Guanaco | 6484643.3 | 348706 | González et al. (2013) | Pelambres | 2000-2011 |
| Guanaco | 6490143.3 | 348706 | González et al. (2013) | Pelambres | 2000-2011 |
| Guanaco | 6490643.3 | 348706 | González et al. (2013) | Pelambres | 2000-2011 |
| Guanaco | 6491143.3 | 348706 | González et al. (2013) | Pelambres | 2000-2011 |
| Guanaco | 6491643.3 | 348706 | González et al. (2013) | Pelambres | 2000-2011 |
| Guanaco | 6492143.3 | 348706 | González et al. (2013) | Pelambres | 2000-2011 |
| Guanaco | 6492643.3 | 348706 | González et al. (2013) | Pelambres | 2000-2011 |
| Guanaco | 6495143.3 | 348706 | González et al. (2013) | Pelambres | 2000-2011 |
| Guanaco | 6486143.3 | 349206 | González et al. (2013) | Pelambres | 2000-2011 |
| Guanaco | 6487143.3 | 349206 | González et al. (2013) | Pelambres | 2000-2011 |
| Guanaco | 6491143.3 | 349206 | González et al. (2013) | Pelambres | 2000-2011 |
| Guanaco | 6491643.3 | 349206 | González et al. (2013) | Pelambres | 2000-2011 |
| Guanaco | 6492143.3 | 349206 | González et al. (2013) | Pelambres | 2000-2011 |
| Guanaco | 6485143.3 | 349706 | González et al. (2013) | Pelambres | 2000-2011 |
| Guanaco | 6491643.3 | 349706 | González et al. (2013) | Pelambres | 2000-2011 |
| Guanaco | 6492143.3 | 349706 | González et al. (2013) | Pelambres | 2000-2011 |
| Guanaco | 6485143.3 | 350206 | González et al. (2013) | Pelambres | 2000-2011 |
| Guanaco | 6492143.3 | 350206 | González et al. (2013) | Pelambres | 2000-2011 |
| Guanaco | 6495143.3 | 350206 | González et al. (2013) | Pelambres | 2000-2011 |
| Guanaco | 6482143.3 | 350706 | González et al. (2013) | Pelambres | 2000-2011 |
| Guanaco | 6490143.3 | 350706 | González et al. (2013) | Pelambres | 2000-2011 |
| Guanaco | 6491643.3 | 350706 | González et al. (2013) | Pelambres | 2000-2011 |
| Guanaco | 6492143.3 | 350706 | González et al. (2013) | Pelambres | 2000-2011 |
| Guanaco | 6493143.3 | 350706 | González et al. (2013) | Pelambres | 2000-2011 |
| Guanaco | 6493643.3 | 350706 | González et al. (2013) | Pelambres | 2000-2011 |
| Guanaco | 6481643.3 | 351206 | González et al. (2013) | Pelambres | 2000-2011 |
| Guanaco | 6490143.3 | 351206 | González et al. (2013) | Pelambres | 2000-2011 |
| Guanaco | 6491143.3 | 351206 | González et al. (2013) | Pelambres | 2000-2011 |
| Guanaco | 6491643.3 | 351206 | González et al. (2013) | Pelambres | 2000-2011 |
| Guanaco | 6490643.3 | 351706 | González et al. (2013) | Pelambres | 2000-2011 |
| Guanaco | 6491143.3 | 351706 | González et al. (2013) | Pelambres | 2000-2011 |
| Guanaco | 6496143.3 | 351706 | González et al. (2013) | Pelambres | 2000-2011 |
| Guanaco | 6496143.3 | 352206 | González et al. (2013) | Pelambres | 2000-2011 |
| Guanaco | 6489643.3 | 352706 | González et al. (2013) | Pelambres | 2000-2011 |
| Guanaco | 6489143.3 | 353206 | González et al. (2013) | Pelambres | 2000-2011 |
| Guanaco | 6490643.3 | 353206 | González et al. (2013) | Pelambres | 2000-2011 |
| Guanaco | 6488643.3 | 353706 | González et al. (2013) | Pelambres | 2000-2011 |
| Guanaco | 6489643.3 | 354206 | González et al. (2013) | Pelambres | 2000-2011 |
| Guanaco | 6490643.3 | 354206 | González et al. (2013) | Pelambres | 2000-2011 |
| Guanaco | 6489643.3 | 354706 | González et al. (2013) | Pelambres | 2000-2011 |
| Guanaco | 6490643.3 | 354706 | González et al. (2013) | Pelambres | 2000-2011 |
| Guanaco | 6492143.3 | 354706 | González et al. (2013) | Pelambres | 2000-2011 |
| Guanaco | 6489643.3 | 355206 | González et al. (2013) | Pelambres | 2000-2011 |
| Guanaco | 6490143.3 | 355206 | González et al. (2013) | Pelambres | 2000-2011 |
| Guanaco | 6491143.3 | 355206 | González et al. (2013) | Pelambres | 2000-2011 |
| Guanaco | 6495643.3 | 355206 | González et al. (2013) | Pelambres | 2000-2011 |
| Guanaco | 6490143.3 | 355706 | González et al. (2013) | Pelambres | 2000-2011 |
| Guanaco | 6491143.3 | 355706 | González et al. (2013) | Pelambres | 2000-2011 |
| Guanaco | 6491643.3 | 355706 | González et al. (2013) | Pelambres | 2000-2011 |
| Guanaco | 6492643.3 | 355706 | González et al. (2013) | Pelambres | 2000-2011 |
| Guanaco | 6489643.3 | 356206 | González et al. (2013) | Pelambres | 2000-2011 |
| Guanaco | 6490143.3 | 356206 | González et al. (2013) | Pelambres | 2000-2011 |
| Guanaco | 6490643.3 | 356206 | González et al. (2013) | Pelambres | 2000-2011 |
| Guanaco | 6489643.3 | 356706 | González et al. (2013) | Pelambres | 2000-2011 |
| Guanaco | 6490143.3 | 356706 | González et al. (2013) | Pelambres | 2000-2011 |
| Guanaco | 6488643.3 | 357206 | González et al. (2013) | Pelambres | 2000-2011 |
| Guanaco | 6490143.3 | 357206 | González et al. (2013) | Pelambres | 2000-2011 |
| Guanaco | 6490643.3 | 357206 | González et al. (2013) | Pelambres | 2000-2011 |
| Guanaco | 6490643.3 | 357706 | González et al. (2013) | Pelambres | 2000-2011 |
| Guanaco | 6492143.3 | 358206 | González et al. (2013) | Pelambres | 2000-2011 |
| Guanaco | 6490643.3 | 358706 | González et al. (2013) | Pelambres | 2000-2011 |
| Guanaco | 6491643.3 | 358706 | González et al. (2013) | Pelambres | 2000-2011 |
| Guanaco | 6490143.3 | 359206 | González et al. (2013) | Pelambres | 2000-2011 |
| Guanaco | 6491143.3 | 359706 | González et al. (2013) | Pelambres | 2000-2011 |
| Guanaco | 6879943.3 | 346206 | Bonacic et al. (2014) | Precordillera | 2014 |
| Guanaco | 6879243.3 | 346906 | Bonacic et al. (2014) | Precordillera | 2014 |
| Guanaco | 6877843.3 | 348306 | Bonacic et al. (2014) | Precordillera | 2014 |
| Guanaco | 6887143.3 | 320206 | Bonacic et al. (2014) | Precordillera | 2014 |
| Guanaco | 6874143.3 | 321706 | Bonacic et al. (2014) | Precordillera | 2014 |
| Guanaco | 6873143.3 | 322206 | Bonacic et al. (2014) | Precordillera | 2014 |
| Guanaco | 6868643.3 | 325706 | González et al. (2013) | Precordillera | 2000-2011 |
| Guanaco | 6864143.3 | 326706 | Researchers contribution | Precordillera | 2008-2012 |
| Guanaco | 6870143.3 | 333706 | Bonacic et al. (2014) | Precordillera | 2014 |
| Guanaco | 6876643.3 | 335206 | Bonacic et al. (2014) | Precordillera | 2014 |
| Guanaco | 6884143.3 | 341206 | Bonacic et al. (2014) | Precordillera | 2014 |
| Guanaco | 6897143.3 | 344706 | Bonacic et al. (2014) | Precordillera | 2014 |
| Guanaco | 6871643.3 | 350706 | Bonacic et al. (2014) | Precordillera | 2014 |
| Guanaco | 6873143.3 | 350706 | Bonacic et al. (2014) | Precordillera | 2014 |
| Guanaco | 6871143.3 | 351206 | Bonacic et al. (2014) | Precordillera | 2014 |
| Guanaco | 6868643.3 | 362706 | Bonacic et al. (2014) | Precordillera | 2014 |
| Guanaco | 6870143.3 | 364206 | Bonacic et al. (2014) | Precordillera | 2014 |
| Guanaco | 6872143.3 | 367206 | Bonacic et al. (2014) | Precordillera | 2014 |
| Guanaco | 6870143.3 | 372706 | Bonacic et al. (2014) | Precordillera | 2014 |
| Guanaco | 6875143.3 | 380206 | Bonacic et al. (2014) | Precordillera | 2014 |
| Guanaco | 6882643.3 | 390206 | Bonacic et al. (2014) | Precordillera | 2014 |
| Guanaco | 6883143.3 | 390206 | Bonacic et al. (2014) | Precordillera | 2014 |
| Guanaco | 6886143.3 | 392706 | Bonacic et al. (2014) | Precordillera | 2014 |
| Guanaco | 7073643.3 | 393206 | Bonacic et al. (2014) | Precordillera | 2014 |
| Guanaco | 6903143.3 | 395206 | González et al. (2013) | Precordillera | 2000-2011 |
| Guanaco | 7079143.3 | 396206 | Bonacic et al. (2014) | Precordillera | 2014 |
| Guanaco | 6890643.3 | 399206 | Bonacic et al. (2014) | Precordillera | 2014 |
| Guanaco | 7045143.3 | 415706 | Bonacic et al. (2014) | Precordillera | 2014 |
| Guanaco | 7043643.3 | 416206 | Bonacic et al. (2014) | Precordillera | 2014 |
| Guanaco | 6735643.3 | 385206 | Collar Satelital_No.121924 | Tres Quebradas River | 2012 - 2015 |
| Guanaco | 6734643.3 | 385706 | Collar Satelital_No.121924 | Tres Quebradas River | 2012 - 2015 |
| Guanaco | 6735143.3 | 385706 | Collar Satelital_No.121924 | Tres Quebradas River | 2012 - 2015 |
| Guanaco | 6735643.3 | 385706 | Collar Satelital_No.121924 | Tres Quebradas River | 2012 - 2015 |
| Guanaco | 6735143.3 | 386206 | Collar Satelital_No.121924 | Tres Quebradas River | 2012 - 2015 |
| Guanaco | 6754143.3 | 389206 | Feces - Sighting | Tres Quebradas River | 2012 - 2014 |
| Guanaco | 6755643.3 | 389706 | Feces - Sighting | Tres Quebradas River | 2012 - 2014 |
| Guanaco | 6753143.3 | 390206 | Feces - Sighting | Tres Quebradas River | 2012 - 2014 |
| Guanaco | 6746143.3 | 390706 | Collar Satelital_No.121924 | Tres Quebradas River | 2012 - 2015 |
| Guanaco | 6746643.3 | 390706 | Collar Satelital_No.121921 | Tres Quebradas River | 2012 - 2015 |
| Guanaco | 6753143.3 | 390706 | Feces - Sighting | Tres Quebradas River | 2012 - 2014 |
| Guanaco | 6755143.3 | 390706 | Collar Satelital_No.121921 | Tres Quebradas River | 2012 - 2015 |
| Guanaco | 6746143.3 | 391206 | Collar Satelital_No.121924 | Tres Quebradas River | 2012 - 2015 |
| Guanaco | 6753143.3 | 391206 | Feces - Sighting | Tres Quebradas River | 2012 - 2014 |
| Guanaco | 6754643.3 | 391206 | Collar Satelital_No.121921 | Tres Quebradas River | 2012 - 2015 |
| Guanaco | 6766143.3 | 391206 | Collar Satelital_No.121921 | Tres Quebradas River | 2012 - 2015 |
| Guanaco | 6737643.3 | 391706 | Collar Satelital_No.121924 | Tres Quebradas River | 2012 - 2015 |
| Guanaco | 6738143.3 | 391706 | Collar Satelital_No.121924 | Tres Quebradas River | 2012 - 2015 |
| Guanaco | 6746143.3 | 391706 | Collar Satelital_No.121924 | Tres Quebradas River | 2012 - 2015 |
| Guanaco | 6753643.3 | 391706 | Feces - Sighting | Tres Quebradas River | 2012 - 2014 |
| Guanaco | 6754143.3 | 391706 | Feces - Sighting | Tres Quebradas River | 2012 - 2014 |
| Guanaco | 6754643.3 | 391706 | Collar Satelital_No.121921 | Tres Quebradas River | 2012 - 2015 |
| Guanaco | 6738643.3 | 392206 | Collar Satelital_No.121924 | Tres Quebradas River | 2012 - 2015 |
| Guanaco | 6753643.3 | 392206 | Feces - Sighting | Tres Quebradas River | 2012 - 2014 |
| Guanaco | 6754143.3 | 392206 | Feces - Sighting | Tres Quebradas River | 2012 - 2014 |
| Guanaco | 6754643.3 | 392206 | Feces - Sighting | Tres Quebradas River | 2012 - 2014 |
| Guanaco | 6753643.3 | 392706 | Feces - Sighting | Tres Quebradas River | 2012 - 2014 |
| Guanaco | 6754643.3 | 392706 | Feces - Sighting | Tres Quebradas River | 2012 - 2014 |
| Guanaco | 6754643.3 | 393206 | Feces - Sighting | Tres Quebradas River | 2012 - 2014 |
| Guanaco | 6769143.3 | 394706 | Collar Satelital_No.121921 | Tres Quebradas River | 2012 - 2015 |
| Guanaco | 6761143.3 | 396206 | Feces - Sighting | Tres Quebradas River | 2012 - 2014 |
| Guanaco | 6761643.3 | 396206 | Feces - Sighting | Tres Quebradas River | 2012 - 2014 |
| Guanaco | 6753643.3 | 396706 | Feces - Sighting | Tres Quebradas River | 2012 - 2014 |
| Guanaco | 6760143.3 | 396706 | Feces - Sighting | Tres Quebradas River | 2012 - 2014 |
| Guanaco | 6757143.3 | 397206 | Collar Satelital_No.121921 | Tres Quebradas River | 2012 - 2015 |
| Guanaco | 6739143.3 | 399206 | Collar Satelital_No.121921 | Tres Quebradas River | 2012 - 2015 |
| Guanaco | 6769143.3 | 399706 | Collar Satelital_No.121921 | Tres Quebradas River | 2012 - 2015 |
| Guanaco | 6740143.3 | 401206 | Collar Satelital_No.121924 | Tres Quebradas River | 2012 - 2015 |
| Guanaco | 6740143.3 | 401706 | Collar Satelital_No.121924 | Tres Quebradas River | 2012 - 2015 |
| Guanaco | 6740643.3 | 401706 | Collar Satelital_No.121924 | Tres Quebradas River | 2012 - 2015 |
| Guanaco | 6739643.3 | 403706 | Collar Satelital_No.121924 | Tres Quebradas River | 2012 - 2015 |
| Guanaco | 6739643.3 | 404206 | Collar Satelital_No.121924 | Tres Quebradas River | 2012 - 2015 |
| Guanaco | 6744143.3 | 404706 | Collar Satelital_No.121924 | Tres Quebradas River | 2012 - 2015 |
| Guanaco | 6744643.3 | 404706 | Collar Satelital_No.121924 | Tres Quebradas River | 2012 - 2015 |
| Guanaco | 6755343.3 | 388206 | Feces - Sighting | Tres Quebradas River | 2012 - 2014 |
| Guanaco | 6756043.3 | 388206 | Feces - Sighting | Tres Quebradas River | 2012 - 2014 |
| Guanaco | 6754643.3 | 388906 | Feces - Sighting | Tres Quebradas River | 2012 - 2014 |
| Guanaco | 6755343.3 | 388906 | Feces - Sighting | Tres Quebradas River | 2012 - 2014 |
| Guanaco | 6753243.3 | 389606 | Feces - Sighting | Tres Quebradas River | 2012 - 2014 |
| Guanaco | 6756043.3 | 389606 | Feces - Sighting | Tres Quebradas River | 2012 - 2014 |
| Guanaco | 6753943.3 | 390306 | Feces - Sighting | Tres Quebradas River | 2012 - 2014 |
| Guanaco | 6754643.3 | 390306 | Feces - Sighting | Tres Quebradas River | 2012 - 2014 |
| Guanaco | 6756043.3 | 390306 | Feces - Sighting | Tres Quebradas River | 2012 - 2014 |
| Guanaco | 6753943.3 | 391006 | Feces - Sighting | Tres Quebradas River | 2012 - 2014 |
| Guanaco | 6755343.3 | 391006 | Feces - Sighting | Tres Quebradas River | 2012 - 2014 |
| Guanaco | 6756043.3 | 391006 | Feces - Sighting | Tres Quebradas River | 2012 - 2014 |
| Guanaco | 6756743.3 | 391006 | Feces - Sighting | Tres Quebradas River | 2012 - 2014 |
| Guanaco | 6763743.3 | 391006 | Feces - Sighting | Tres Quebradas River | 2012 - 2014 |
| Guanaco | 6767243.3 | 391006 | Feces - Sighting | Tres Quebradas River | 2012 - 2014 |
| Guanaco | 6755343.3 | 391706 | Feces - Sighting | Tres Quebradas River | 2012 - 2014 |
| Guanaco | 6763743.3 | 391706 | Feces - Sighting | Tres Quebradas River | 2012 - 2014 |
| Guanaco | 6764443.3 | 391706 | Feces - Sighting | Tres Quebradas River | 2012 - 2014 |
| Guanaco | 6765143.3 | 391706 | Feces - Sighting | Tres Quebradas River | 2012 - 2014 |
| Guanaco | 6766543.3 | 391706 | Feces - Sighting | Tres Quebradas River | 2012 - 2014 |
| Guanaco | 6767243.3 | 391706 | Feces - Sighting | Tres Quebradas River | 2012 - 2014 |
| Guanaco | 6767943.3 | 391706 | Feces - Sighting | Tres Quebradas River | 2012 - 2014 |
| Guanaco | 6768643.3 | 391706 | Feces - Sighting | Tres Quebradas River | 2012 - 2014 |
| Guanaco | 6769343.3 | 391706 | Feces - Sighting | Tres Quebradas River | 2012 - 2014 |
| Guanaco | 6753243.3 | 392406 | Feces - Sighting | Tres Quebradas River | 2012 - 2014 |
| Guanaco | 6755343.3 | 392406 | Feces - Sighting | Tres Quebradas River | 2012 - 2014 |
| Guanaco | 6756043.3 | 392406 | Feces - Sighting | Tres Quebradas River | 2012 - 2014 |
| Guanaco | 6763743.3 | 392406 | Feces - Sighting | Tres Quebradas River | 2012 - 2014 |
| Guanaco | 6764443.3 | 392406 | Feces - Sighting | Tres Quebradas River | 2012 - 2014 |
| Guanaco | 6765843.3 | 392406 | Feces - Sighting | Tres Quebradas River | 2012 - 2014 |
| Guanaco | 6766543.3 | 392406 | Feces - Sighting | Tres Quebradas River | 2012 - 2014 |
| Guanaco | 6767243.3 | 392406 | Feces - Sighting | Tres Quebradas River | 2012 - 2014 |
| Guanaco | 6767943.3 | 392406 | Feces - Sighting | Tres Quebradas River | 2012 - 2014 |
| Guanaco | 6769343.3 | 392406 | Feces - Sighting | Tres Quebradas River | 2012 - 2014 |
| Guanaco | 6753243.3 | 393106 | Feces - Sighting | Tres Quebradas River | 2012 - 2014 |
| Guanaco | 6756043.3 | 393106 | Feces - Sighting | Tres Quebradas River | 2012 - 2014 |
| Guanaco | 6756743.3 | 393106 | Feces - Sighting | Tres Quebradas River | 2012 - 2014 |
| Guanaco | 6763743.3 | 393106 | Feces - Sighting | Tres Quebradas River | 2012 - 2014 |
| Guanaco | 6767243.3 | 393106 | Feces - Sighting | Tres Quebradas River | 2012 - 2014 |
| Guanaco | 6768643.3 | 393106 | Feces - Sighting | Tres Quebradas River | 2012 - 2014 |
| Guanaco | 6769343.3 | 393106 | Feces - Sighting | Tres Quebradas River | 2012 - 2014 |
| Guanaco | 6754643.3 | 393806 | Feces - Sighting | Tres Quebradas River | 2012 - 2014 |
| Guanaco | 6755343.3 | 393806 | Feces - Sighting | Tres Quebradas River | 2012 - 2014 |
| Guanaco | 6763043.3 | 393806 | Feces - Sighting | Tres Quebradas River | 2012 - 2014 |
| Guanaco | 6763743.3 | 393806 | Feces - Sighting | Tres Quebradas River | 2012 - 2014 |
| Guanaco | 6766543.3 | 393806 | Feces - Sighting | Tres Quebradas River | 2012 - 2014 |
| Guanaco | 6769343.3 | 393806 | Feces - Sighting | Tres Quebradas River | 2012 - 2014 |
| Guanaco | 6753943.3 | 394506 | Feces - Sighting | Tres Quebradas River | 2012 - 2014 |
| Guanaco | 6754643.3 | 394506 | Feces - Sighting | Tres Quebradas River | 2012 - 2014 |
| Guanaco | 6755343.3 | 394506 | Feces - Sighting | Tres Quebradas River | 2012 - 2014 |
| Guanaco | 6760243.3 | 394506 | Feces - Sighting | Tres Quebradas River | 2012 - 2014 |
| Guanaco | 6763043.3 | 394506 | Feces - Sighting | Tres Quebradas River | 2012 - 2014 |
| Guanaco | 6764443.3 | 394506 | Feces - Sighting | Tres Quebradas River | 2012 - 2014 |
| Guanaco | 6765843.3 | 394506 | Feces - Sighting | Tres Quebradas River | 2012 - 2014 |
| Guanaco | 6766543.3 | 394506 | Feces - Sighting | Tres Quebradas River | 2012 - 2014 |
| Guanaco | 6767943.3 | 394506 | Feces - Sighting | Tres Quebradas River | 2012 - 2014 |
| Guanaco | 6770043.3 | 394506 | Feces - Sighting | Tres Quebradas River | 2012 - 2014 |
| Guanaco | 6754643.3 | 395206 | Feces - Sighting | Tres Quebradas River | 2012 - 2014 |
| Guanaco | 6758143.3 | 395206 | Feces - Sighting | Tres Quebradas River | 2012 - 2014 |
| Guanaco | 6758843.3 | 395206 | Feces - Sighting | Tres Quebradas River | 2012 - 2014 |
| Guanaco | 6760243.3 | 395206 | Feces - Sighting | Tres Quebradas River | 2012 - 2014 |
| Guanaco | 6760943.3 | 395206 | Feces - Sighting | Tres Quebradas River | 2012 - 2014 |
| Guanaco | 6761643.3 | 395206 | Feces - Sighting | Tres Quebradas River | 2012 - 2014 |
| Guanaco | 6762343.3 | 395206 | Feces - Sighting | Tres Quebradas River | 2012 - 2014 |
| Guanaco | 6765143.3 | 395206 | Feces - Sighting | Tres Quebradas River | 2012 - 2014 |
| Guanaco | 6766543.3 | 395206 | Feces - Sighting | Tres Quebradas River | 2012 - 2014 |
| Guanaco | 6769343.3 | 395206 | Feces - Sighting | Tres Quebradas River | 2012 - 2014 |
| Guanaco | 6757443.3 | 395906 | Feces - Sighting | Tres Quebradas River | 2012 - 2014 |
| Guanaco | 6758143.3 | 395906 | Feces - Sighting | Tres Quebradas River | 2012 - 2014 |
| Guanaco | 6758843.3 | 395906 | Feces - Sighting | Tres Quebradas River | 2012 - 2014 |
| Guanaco | 6759543.3 | 395906 | Feces - Sighting | Tres Quebradas River | 2012 - 2014 |
| Guanaco | 6760943.3 | 395906 | Feces - Sighting | Tres Quebradas River | 2012 - 2014 |
| Guanaco | 6763043.3 | 395906 | Feces - Sighting | Tres Quebradas River | 2012 - 2014 |
| Guanaco | 6768643.3 | 395906 | Feces - Sighting | Tres Quebradas River | 2012 - 2014 |
| Guanaco | 6756743.3 | 396606 | Feces - Sighting | Tres Quebradas River | 2012 - 2014 |
| Guanaco | 6758143.3 | 396606 | Feces - Sighting | Tres Quebradas River | 2012 - 2014 |
| Guanaco | 6758843.3 | 396606 | Feces - Sighting | Tres Quebradas River | 2012 - 2014 |
| Guanaco | 6763043.3 | 396606 | Feces - Sighting | Tres Quebradas River | 2012 - 2014 |
| Guanaco | 6763743.3 | 396606 | Feces - Sighting | Tres Quebradas River | 2012 - 2014 |
| Guanaco | 6766543.3 | 396606 | Feces - Sighting | Tres Quebradas River | 2012 - 2014 |
| Guanaco | 6769343.3 | 396606 | Feces - Sighting | Tres Quebradas River | 2012 - 2014 |
| Guanaco | 6758143.3 | 397306 | Feces - Sighting | Tres Quebradas River | 2012 - 2014 |
| Guanaco | 6760943.3 | 397306 | Feces - Sighting | Tres Quebradas River | 2012 - 2014 |
| Guanaco | 6762343.3 | 397306 | Feces - Sighting | Tres Quebradas River | 2012 - 2014 |
| Guanaco | 6763043.3 | 397306 | Feces - Sighting | Tres Quebradas River | 2012 - 2014 |
| Guanaco | 6764443.3 | 397306 | Feces - Sighting | Tres Quebradas River | 2012 - 2014 |
| Guanaco | 6767943.3 | 397306 | Feces - Sighting | Tres Quebradas River | 2012 - 2014 |
| Guanaco | 6759543.3 | 398006 | Feces - Sighting | Tres Quebradas River | 2012 - 2014 |
| Guanaco | 6760243.3 | 398006 | Feces - Sighting | Tres Quebradas River | 2012 - 2014 |
| Guanaco | 6761643.3 | 398006 | Feces - Sighting | Tres Quebradas River | 2012 - 2014 |
| Guanaco | 6762343.3 | 398006 | Feces - Sighting | Tres Quebradas River | 2012 - 2014 |
| Guanaco | 6763743.3 | 398006 | Feces - Sighting | Tres Quebradas River | 2012 - 2014 |
| Guanaco | 6765143.3 | 398006 | Feces - Sighting | Tres Quebradas River | 2012 - 2014 |
| Guanaco | 6765843.3 | 398006 | Feces - Sighting | Tres Quebradas River | 2012 - 2014 |
| Guanaco | 6766543.3 | 398006 | Feces - Sighting | Tres Quebradas River | 2012 - 2014 |
| Guanaco | 6760243.3 | 398706 | Feces - Sighting | Tres Quebradas River | 2012 - 2014 |
| Guanaco | 6761643.3 | 398706 | Feces - Sighting | Tres Quebradas River | 2012 - 2014 |
| Guanaco | 6763743.3 | 398706 | Feces - Sighting | Tres Quebradas River | 2012 - 2014 |
| Guanaco | 6770043.3 | 398706 | Feces - Sighting | Tres Quebradas River | 2012 - 2014 |
| Guanaco | 6761643.3 | 399406 | Feces - Sighting | Tres Quebradas River | 2012 - 2014 |
| Guanaco | 6763743.3 | 399406 | Feces - Sighting | Tres Quebradas River | 2012 - 2014 |
| Guanaco | 6764443.3 | 399406 | Feces - Sighting | Tres Quebradas River | 2012 - 2014 |
| Guanaco | 6765143.3 | 399406 | Feces - Sighting | Tres Quebradas River | 2012 - 2014 |
| Guanaco | 6767943.3 | 399406 | Feces - Sighting | Tres Quebradas River | 2012 - 2014 |
| Guanaco | 6762343.3 | 400106 | Feces - Sighting | Tres Quebradas River | 2012 - 2014 |
| Guanaco | 6763043.3 | 400106 | Feces - Sighting | Tres Quebradas River | 2012 - 2014 |
| Guanaco | 6763743.3 | 400106 | Feces - Sighting | Tres Quebradas River | 2012 - 2014 |
| Guanaco | 6764443.3 | 400106 | Feces - Sighting | Tres Quebradas River | 2012 - 2014 |
| Guanaco | 6770043.3 | 400106 | Feces - Sighting | Tres Quebradas River | 2012 - 2014 |
| Guanaco | 6761643.3 | 400806 | Feces - Sighting | Tres Quebradas River | 2012 - 2014 |
| Guanaco | 6762343.3 | 400806 | Feces - Sighting | Tres Quebradas River | 2012 - 2014 |
| Guanaco | 6763043.3 | 400806 | Feces - Sighting | Tres Quebradas River | 2012 - 2014 |
| Guanaco | 6763743.3 | 400806 | Feces - Sighting | Tres Quebradas River | 2012 - 2014 |
| Guanaco | 6761643.3 | 401506 | Feces - Sighting | Tres Quebradas River | 2012 - 2014 |
| Guanaco | 6761643.3 | 402206 | Feces - Sighting | Tres Quebradas River | 2012 - 2014 |
| Guanaco | 6762829.5 | 398689.2 | Feces - Sighting | Tres Quebradas River | 2012 - 2014 |
| Guanaco | 6762143.3 | 394706 | Feces - Sighting | Tres Quebradas River | 2012 - 2014 |
| Guanaco | 6758143.3 | 397706 | Feces - Sighting | Tres Quebradas River | 2012 - 2014 |

**References**

Bonacic C, Bonacic D, Muñoz A, Riveros J, Vargas S, Soto J. 2014. Estrategia multisectorial para la conservación de camélidos silvestres sudamericanos de la Región de Atacama. Chile: Laboratorio Fauna Australis, Facultad de Agronomía e Ingeniería Forestal, Pontificia Universidad Católica de Chile, 52.

González BA, Samaniego H, Marín JC, Estades CF. 2013. Unveiling current guanaco distribution in Chile based upon niche structure of phylogeographic lineages: Andean Puna to subpolar forests. *PLOS ONE* 8:e78894 DOI 10.1371/journal.pone.0078894.

SEIA. 1994. Estudio de impacto ambiental. Proyecto Minero El Tambo. Chile, Región de Coquimbo: Sistema de Evaluación de Impacto Ambiental. *Available at* [*http://www.sea.gob.cl/*](http://www.sea.gob.cl/) (accessed on the 10^th^ Feb 2016).

SEIA. 2011. Estudio de impacto ambiental. Explotación Minera El Morro. Chile, Región de Atacama: Sistema de Evaluación de Impacto Ambiental. *Available at* [*http://www.sea.gob.cl/*](http://www.sea.gob.cl/) (accessed on the 10^th^ Feb 2016).
