# Supplementary material for: Landscape connectivity among remnant populations of guanaco (Lama guanicoe Müller, 1776) in an arid region of Chile impacted by global change
Source: PeerJ. 2018 Mar 2;6:e4429. doi: 10.7717/peerj.4429 (PMC5836568; doi:10.7717/peerj.4429)
Supplement: Table S2 — Source: (Luebert & Pliscoff, 2006). [file peerj-06-4429-s002.docx]

| **No.** | **Vegetation communities** | **Formation** |
| --- | --- | --- |
| 1 | Andean Mediterranean sclerophyll forest of *Kageneckia angustifolia* and Guindilia trinervis | Sclerophyll forest |
| 2 | Mediterranean Coastal sclerophyll forest of *Cryptocarya alba* and *Peumus boldus* | Sclerophyll forest |
| 3 | Mediterranean Coastal sclerophyll forest of *Lithrea caustica* and *Cryptocarya alba* | Sclerophyll forest |
| 4 | Mediterranean pastureland of *Nastanthus spathulatus* and *Menonvillea spathulata* | High altitude grassland |
| 5 | Andean tropical pastureland of *Chaetanthera sphaeroidalis* | High altitude grassland |
| 6 | Mediterranean Coastal sclerophyllous arborescent shrubland of *Peumus boldus* and *Schinus latifolius* | Arborescent shrubland |
| 7 | Interior Mediterranean sclerophyllous Arborescent shrubland *Quillaja saponaria* and *Porlieria chilens* | Arborescent shrubland |
| 8 | Andean Mediterranean Desert underbrush of *Senecio proteus* and *Haplopappus baylahuen* | Desert underbrush |
| 9 | Andean tropical Mediterranean Desert underbrush of *Atriplex imbricata* | Desert underbrush |
| 10 | Interior tropical Desert underbrush of *Adesmia atacamensis* and *Cistanthe salsoloides* | Desert underbrush |
| 11 | Interior tropical Desert underbrush of *Nolana leptophylla* and *Cistanthe salsoloides* | Desert underbrush |
| 12 | Andean tropical Mediterranean underbrush of *Chuquiraga oppositifolia* and *Nardophyllum lanatum* | Andean underbrush |
| 13 | Andean Mediterranean underbrush of *Laretia acaulis* and *Berberis empetrifolia* | Andean underbrush |
| 14 | Andean tropical Mediterranean underbrush of *Adesmia hystrix* and *Ephedra breana* | Andean underbrush |
| 15 | Andean Mediterranean underbrush *Adesmia subterranea* and *Adesmia echinus* | Andean underbrush |
| 16 | Andean tropical underbrush of *Adesmia frigida* and *Stipa frigida* | Andean underbrush |
| 17 | Andean tropical underbrush of *Artemisia* copa and *Stipa frigida* | Andean underbrush |
| 18 | Andean tropical underbrush of *Fabiana bryoides* and *Parastrephia quadrangularis* | Andean underbrush |
| 19 | Andean tropical underbrush of *Mulinum crassifolium* and *Urbania pappigera* | Andean underbrush |
| 20 | Mediterranean Coastal Desert Thicket of *Bahia ambrosioides* and *Puya chilensis* | Desert shrubland |
| 21 | Mediterranean Coastal Desert Thicket of *Euphorbia lactiflua* and *Eulychnia saint-pieana* | Desert shrubland |
| 22 | Mediterranean Coastal Desert Thicket of *Gypothamnium pinifolium* and *Heliotropium pycnophyllu* | Desert shrubland |
| 23 | Mediterranean Coastal Desert Thicket of *Heliotropium floridum* and *Atriplex clivicola* | Desert shrubland |
| 24 | Mediterranean Coastal Desert Thicket of *Oxalis gigantea* and *Eulychnia breviflora* | Desert shrubland |
| 25 | Mediterranean Coastal Desert Thicket of *Oxalis gigantea* and *Heliotropium stenophyllum* | Desert shrubland |
| 26 | Mediterranean Interior Desert scrubland of Adesmia argentea and Bulnesia chilensis | Desert shrubland |
| 27 | Mediterranean Interior Desert scrubland of *Flourensia thurifera* and *Colliguaja odorifera* | Desert shrubland |
| 28 | Mediterranean Interior Desert scrubland of *Heliotropium stenophyllum* and *Flourensia thurifera* | Desert shrubland |
| 29 | Mediterranean Interior Desert scrubland of *Oxyphyllum ulicinum* and *Gymnophyton foliosum* | Desert shrubland |
| 30 | Mediterranean Interior Desert scrubland of *Skytanthus acutus* and *Atriplex deserticola* | Desert shrubland |
| 31 | Interior tropical Desert scrubland of *Huidobria* *chilensis* and *Nolana leptophylla* | Desert shrubland |
| 32 | Interior Mediterranean thorny shrubland of *Puya coerulea* and *Colliguaja odorifera* | Thorny shrubland |
| 33 | Interior Mediterranean thorny shrubland of *Trevoa quinquinervia* and *Colliguaja odorifera* | Thorny shrubland |

Supplementary Table S2

Vegetation communities used for habitat suitability modelling of *Lama guanicoe* in Chile’s Norte Chico. Source: (Luebert & Pliscoff, 2006)
