# Supplementary material for: Landscape connectivity among remnant populations of guanaco (Lama guanicoe Müller, 1776) in an arid region of Chile impacted by global change
Source: PeerJ. 2018 Mar 2;6:e4429. doi: 10.7717/peerj.4429 (PMC5836568; doi:10.7717/peerj.4429)
Supplement: Table S3 [file peerj-06-4429-s003.docx]

Supplementary Table S3

Food items listed according to species and vegetation communities contributing strongly to *Lama guanicoe* distribution in the study area

| **No.** | **Vegetation communities** | **Known Food item** | **Source** |
| --- | --- | --- | --- |
| 1 | Andean Mediterranean Sclerophyll Forest | *Nasella chilensis* | Study of Guanaco diet in Los Choros stream, Chile. Andes Iron 2013. Environmental impact assessment (SEIA, 2013). |
| 1 | Andean Mediterranean Sclerophyll Forest | *Viviania marifolia* | Census, habitat use and trophic ecology of Guanacos in Estrecho River, Fall 2011. La Serena University - Pascua-Lama Mining Project. Unpublished data |
| 4 | Mediterranean Pastureland | *Oxychloe andina* | Census, habitat use and trophic ecology of Guanacos in Estrecho River, Fall 2011. La Serena University - Pascua-Lama Mining Project. Unpublished data |
| 4 | Mediterranean Pastureland | *Deyeuxia eminens* | Study of Guanaco diet in the Atacama Region - La Serena University. Reports from Environmental Impact Study, El Morro Mining Company, (SEIA, 2011). |
| 4 | Mediterranean Pastureland | *Deyeuxia velutina* | Study of Guanaco diet in the Atacama Region - La Serena University. Reports from Environmental Impact Study, El Morro Mining Company, (SEIA, 2011). |
| 12 | Andean Mediterranean underbrush | *Junellia sp.* | Census, habitat use and trophic ecology of Guanacos in Estrecho River, Summer 2011. La Serena University - Pascua-Lama Mining Project. Unpublished data |
| 13 | Andean tropical Mediterranean underbrush | *Junellia uniflora* | Census, habitat use and trophic ecology of Guanacos in Estrecho River, Summer 2011. La Serena University - Pascua-Lama Mining Project. Unpublished data |
| 24 | Mediterranean Coastal Desert Thicket | *Frankenia chilensis* | Study of Guanaco diet in Los Choros, Chile.  Andes Iron 2013. Environmental impact assessment (SEIA, 2013) |
| 24 | Mediterranean Coastal Desert Thicket | *Nolana divaricata* | Study of Guanaco diet in Los Choros, Chile.  Andes Iron 2013. Environmental impact assessment (SEIA, 2013) |
| 24 | Mediterranean Coastal Desert Thicket | *Cristaria aspera* | Study of Guanaco diet in Los Choros, Chile.  Andes Iron 2013. Environmental impact assessment (SEIA, 2013) |
| 24 | Mediterranean Coastal Desert Thicket | *Ophryosporus triangularis* | Study of Guanaco diet in Los Choros, Chile.  Andes Iron 2013. Environmental impact assessment (SEIA, 2013) |
| 25 | Mediterranean Coastal Desert Thicket | *Encelia canescens* | Study of Guanaco diet in Los Choros, Chile.  Andes Iron 2013. Environmental impact assessment (SEIA, 2013) |
| 25 | Mediterranean Coastal Desert Thicket | *Haplopappus sp* | Study of Guanaco diet in Los Choros, Chile.  Andes Iron 2013. Environmental impact assessment (SEIA, 2013) |
| 25 | Mediterranean Coastal Desert Thicket | *Flourencia thurifera* | Study of Guanaco diet in Los Choros, Chile.  Andes Iron 2013. Environmental impact assessment (SEIA, 2013) |
| 25 | Mediterranean Coastal Desert Thicket | *Mesembryantemum cristallinum* | Study of Guanaco diet in Los Choros, Chile.  Andes Iron 2013. Environmental impact assessment (SEIA, 2013) |
| 28 | Mediterranean interior desert scrubland | *Adesmia microphylla* | Study of Guanaco diet in Los Choros, Chile.  Andes Iron 2013. Environmental impact assessment (SEIA, 2013) |
| 28 | Mediterranean interior desert scrubland | *Oxalis perdicaria* | Study of Guanaco diet in Los Choros, Chile.  Andes Iron 2013. Environmental impact assessment (SEIA, 2013) |
| 28 | Mediterranean interior desert scrubland | *Encelia canescens* | Study of Guanaco diet in Los Choros, Chile.  Andes Iron 2013. Environmental impact assessment (SEIA, 2013) |
| 28 | Mediterranean interior desert scrubland | *Haplopappus sp* | Study of Guanaco diet in Los Choros, Chile.  Andes Iron 2013. Environmental impact assessment (SEIA, 2013) |
| 28 | Mediterranean interior desert scrubland | *Oxalis virgosa* | Study of Guanaco diet in Los Choros, Chile.  Andes Iron 2013. Environmental impact assessment (SEIA, 2013) |
| 28 | Mediterranean interior desert scrubland | *Flourencia thurifera* | Study of Guanaco diet in Los Choros, Chile.  Andes Iron 2013. Environmental impact assessment (SEIA, 2013) |
| 28 | Mediterranean interior desert scrubland | *Pleocarpus revolutus* | Study of Guanaco diet in Los Choros, Chile.  Andes Iron 2013. Environmental impact assessment (SEIA, 2013) |
| 28 | Mediterranean interior desert scrubland | *Ophryosporus triangularis* | Study of Guanaco diet in Los Choros, Chile.  Andes Iron 2013. Environmental impact assessment (SEIA, 2013) |

**References**

SEIA. 2013. Estudio de impacto ambiental. Explotación Minera Dominga. Chile, Región de Atacama: Sistema de Evaluación de Impacto Ambiental. *Available at* [*http://www.sea.gob.cl/*](http://www.sea.gob.cl/) (accessed on the 10^th^ Feb 2016).

SEIA. 2011. Estudio de impacto ambiental. Explotación Minera El Morro. Chile, Región de Atacama: Sistema de Evaluación de Impacto Ambiental. *Available at* [*http://www.sea.gob.cl/*](http://www.sea.gob.cl/) (accessed on the 10^th^ Feb 2016).
