# Supplementary material for: Landscape connectivity among remnant populations of guanaco (Lama guanicoe Müller, 1776) in an arid region of Chile impacted by global change
Source: PeerJ. 2018 Mar 2;6:e4429. doi: 10.7717/peerj.4429 (PMC5836568; doi:10.7717/peerj.4429)
Supplement: Table S4 [file peerj-06-4429-s004.docx]

Supplementary Table S4

Extinction risk level of the *Lama guanicoe* populations in Chile’s Norte Chico based on potential population size, local threats and connectivity extent approximated by current flow centrality values.

| **ID** | **Habitat patch name** | **Estimated population size** | **Protection status** | **Local threats** | **Current flow centrality** | **References** |
| --- | --- | --- | --- | --- | --- | --- |
| 1 | Pan de Azúcar National Park | 120 | National park | Poaching/ Dog attacks | 11.8 | Census of Pan de Azúcar National park. Historical average from 2000-2015. Professional reports, (CONAF, 2015). |
| 2 | Nevado Tres Cruces National Park | 80 | National Park | Poaching/ Dog attacks/ Disease by scabies | 15.9 | Census of Nevado Tres cruces National park. Historical average from 2007-2016. Professional reports, (CONAF, 2016). |
| 3 | Llanos de Challe National Park | 900 | National Park | Poaching/dog attacks/ Vehicle collisions | 15.2 | Guanaco’s Conservation national plan 2010-2015. (CONAF, 2010) |
| 4 | Oso Negro sector | 70 | Flowering desert priority site | Poaching/ Vehicle collisions | 23.7 | Reports from Environmental Impact Study, Oso Negro Mining Company, (SEIA, 2012).  González & Acebes (2016) |
| 5 | El Morro | 200 | Private Protected Area | Poaching/ Habitat loss | 37.1 | Reports from Environmental Impact Study, El Morro Mining Company, (SEIA, 2011).  Opinion of Expert (Cortes A. Universidad de La Serena, Pers. Com.) |
| 6 | Los Choros | 300 | Unprotected area | Dog attacks/ Vehicle collisions/ Competition with livestock | 13.6 | Yearly Census of Guanacos. Andes Iron 2012 – 2013. Environmental Impact Study (SEIA, 2013). |
| 7 | Calvario stream | 200 | Unprotected area | Poaching/ Competition with livestock | 29.3 | Pascua-Lama Mining Project, Biodiversity Report 2012. Unpublished data: Seasonal census 2012-2014. |
| 8 | Tres Quebradas River | 500 | High Conservation Value Area | Poaching/ Habitat loss | 24.2 | Pascua-Lama Mining Project, Biodiversity Report 2012. Unpublished data: Seasonal census 2012-2014. Opinion of Expert (Cortes A. Universidad de La Serena, Pers. Com.) |
| 9 | El Tambo stream | 50 | High Conservation Value Area | Poaching | 20.5 | Reports from Environmental Impact Study, El Tambo Mining Company (SEIA, 1994).  Opinion of Expert (Cortes A., Osorio R., Universidad de La Serena, Pers. Com. Seasonal census 2002-2008). |
| 10 | Estero Derecho nature sanctuary | 100 | Private Protected Area & Nature Sanctuary | Competition with livestock | 20.9 | Management plan of Estero derecho, (APP-SN, 2016); Opinion of Expert Osorio R., Universidad de La Serena, Pers. Com. |
| 11 | Pelambres Area | 1300 | Unprotected area / Private area of Pelambres Mining Company | Habitat loss | 10 | González & Acebes (2016) |

**References**

APP-SN. Área Protegida Privada y Santuario de la Naturaleza ‘‘Estero Derecho’’. 2016. Plan de Manejo de Estero Derecho. Available at <http://www.esteroderecho.cl/> (accessed on 24 May 2017).

CONAF. 2010. Plan Nacional de Conservación del Guanaco (Lama guanicoe, Müller, 1776) en Chile, 2010–2015. Macrozona Norte y Centro. Chile: Corporación Nacional Forestal. *Available at* [*http://www.conaf.cl/*](http://www.conaf.cl/) (accessed on the 20^th^ April 2014)

CONAF. 2015. Censos de Guanacos (Lama guanicoe) Parque Nacional Pan de Azúcar. 2000–2015. Chile, Región de Atacama: Corporación Nacional Forestal.

CONAF. 2016. Censos de Guanacos (Lama guanicoe) Parque Nacional Nevado Tres Cruces. 2007–2016. Chile, Región de Atacama: Corporación Nacional Forestal.

González BA, Acebes P. 2016. Reevaluación del guanaco para la Lista Roja de la IUCN: situación actual y recomendaciones a futuro. Switzerland: GECS News **6**:15–21.

SEIA. Sistema de Evaluación de Impacto Ambiental. 2011. Estudio de impacto ambiental. Explotación Minera El Morro. Chile, Región de Atacama: Sistema de Evaluación de Impacto Ambiental. *Available at* [*http://www.sea.gob.cl/*](http://www.sea.gob.cl/) (accessed on the 10^th^ Feb 2016).

SEIA. 2011. Estudio de impacto ambiental. Explotación Minera El Morro. Chile, Región de Atacama: Sistema de Evaluación de Impacto Ambiental. *Available at* [*http://www.sea.gob.cl/*](http://www.sea.gob.cl/) (accessed on the 10^th^ Feb 2016).

SEIA. 2012. Estudio de impacto ambiental. Explotación Minera Oso Negro. Chile, Región de Atacama: Sistema de Evaluación de Impacto Ambiental. *Available at* [*http://www.sea.gob.cl/*](http://www.sea.gob.cl/) (accessed on the 10^th^ Feb 2016).

SEIA. Sistema de Evaluación de Impacto Ambiental. 2013. Estudio de impacto ambiental. Explotación Minera Dominga. Chile, Región de Atacama: Sistema de Evaluación de Impacto Ambiental. *Available at* [*http://www.sea.gob.cl/*](http://www.sea.gob.cl/) (accessed on the 10^th^ Feb 2016).
