# Supplementary figures and images for: Landscape connectivity among remnant populations of guanaco (Lama guanicoe Müller, 1776) in an arid region of Chile impacted by global change
Source: PeerJ. 2018 Mar 2;6:e4429. doi: 10.7717/peerj.4429 (PMC5836568; doi:10.7717/peerj.4429)

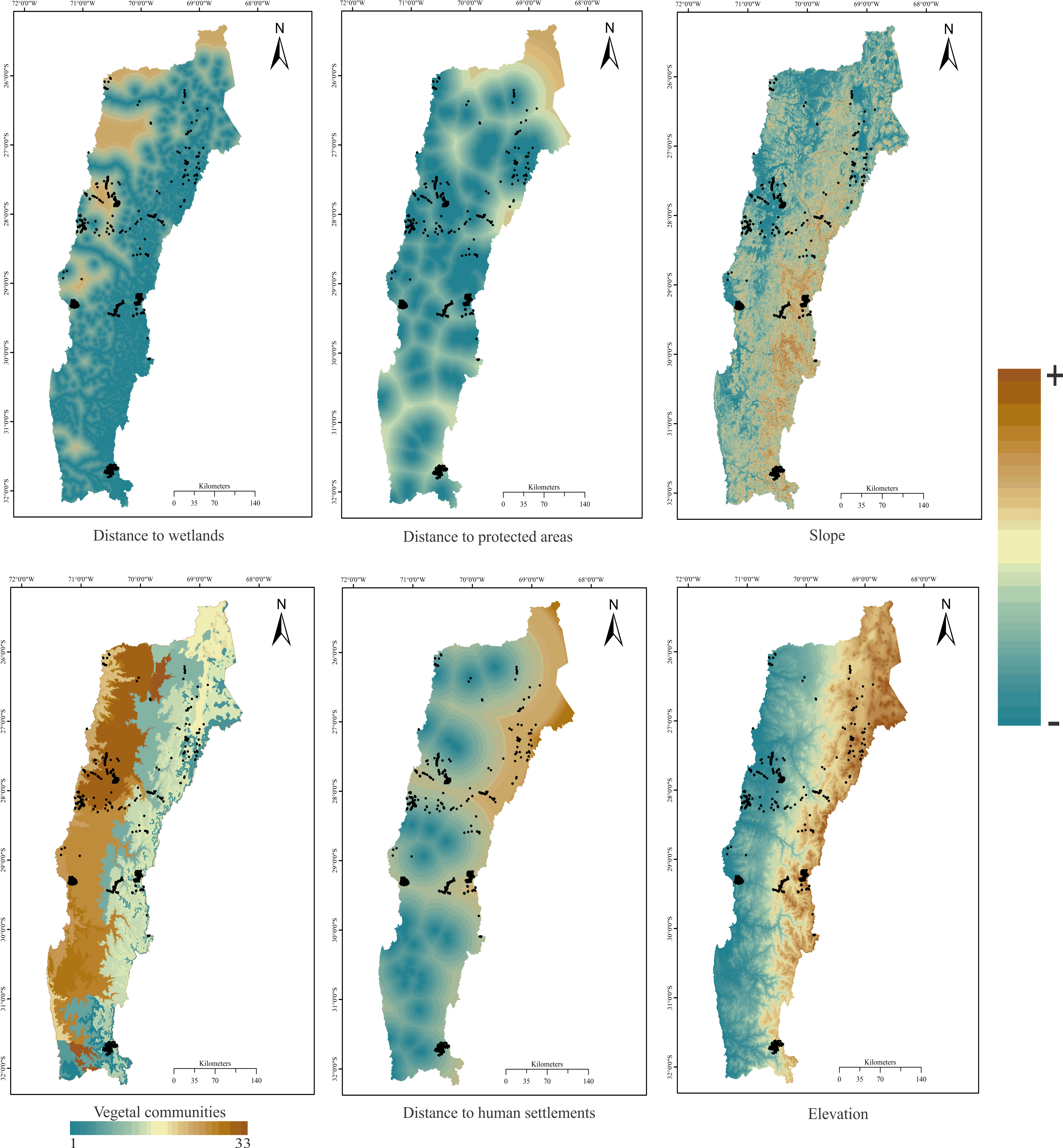

Supplement: Figure S1 — (see Methodology). [file peerj-06-4429-s005.png]

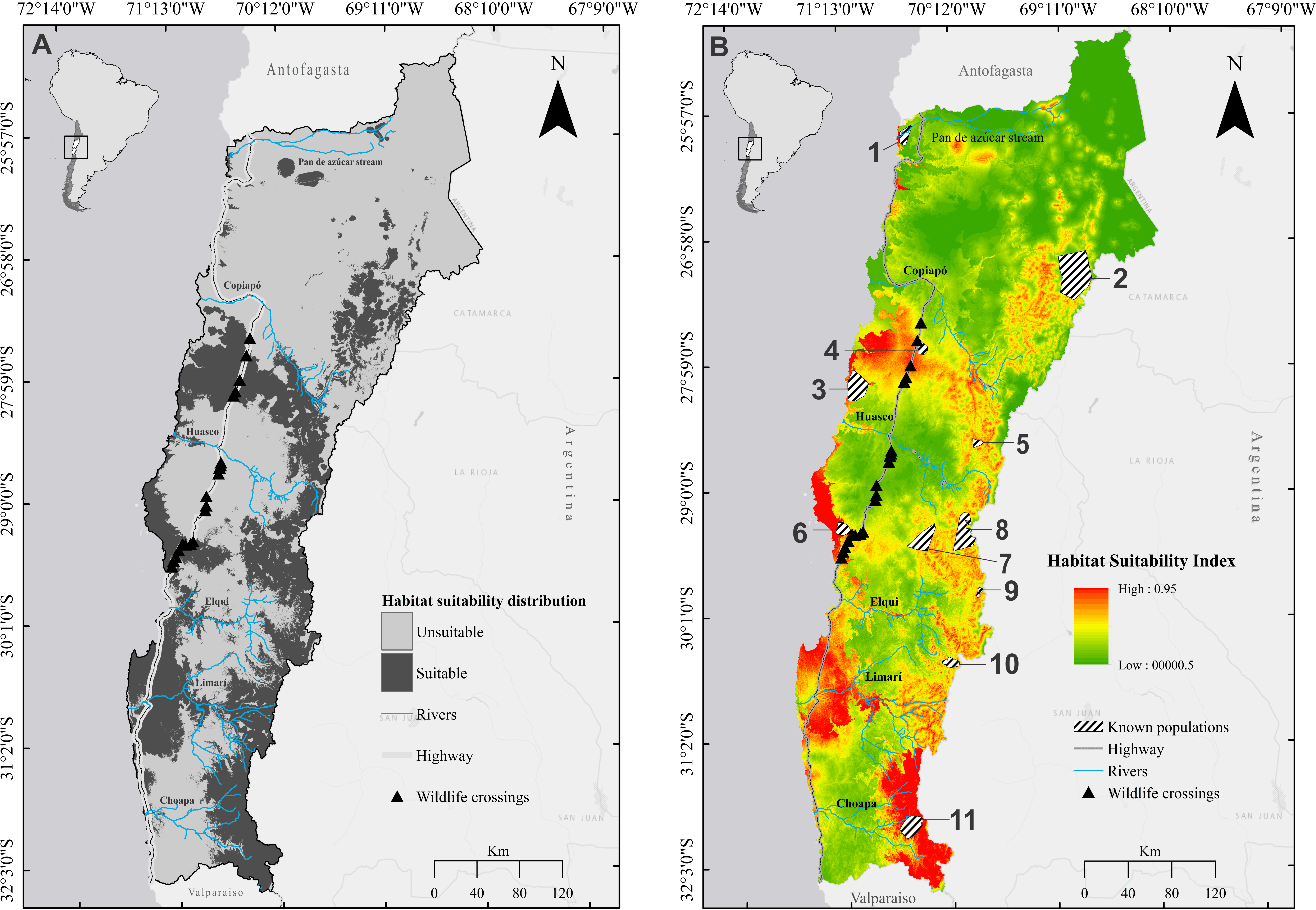

Supplement: Figure S2 — The suitable habitat threshold was defined as the habitat suitability index value that maximized the sum of sensitivity and specificity (see Methodology). Habitat patches corresponding to the guanaco populations are represented in Figure part B: 1, Pan de Azúcar National Park; 2, Nevado Tres Cruces National Park; 3, Llanos de Challe National Park; 4, Oso Negro sector; 5, El Morro; 6, Los Choros; 7, Calvario stream; 8, Tres Quebradas river; 9, El Tambo stream; 10, Estero Derecho nature sanctuary; 11, Pelambres area. [file peerj-06-4429-s006.png]
